# Fully Exploited Oxygen Redox Reaction by the Inter‐Diffused Cations in Co‐Free Li‐Rich Materials for High Performance Li‐Ion Batteries

**DOI:** 10.1002/advs.202001658

**Published:** 2020-07-20

**Authors:** Junghwa Lee, Nicolas Dupre, Mihee Jeong, ShinYoung Kang, Maxim Avdeev, Yue Gong, Lin Gu, Won‐Sub Yoon, Byoungwoo Kang

**Affiliations:** ^1^ Department of Materials Science and Engineering Pohang University of Science and Technology (POSTECH) Pohang 37673 Republic of Korea; ^2^ Institut des Materiaux Jean Rouxel (IMN) Université de Nantes CNRS UMR 6502, 2 rue de la Houssiniere, BP 32229 Nantes Cedex 3 44322 France; ^3^ Department of Energy Science Sungkyunkwan University Suwon 16419 Republic of Korea; ^4^ Lawrence Livermore National Laboratory 7000 East Avenue, L‐413 Livermore CA 94550 US; ^5^ Australian Nuclear Science and Technology Organization Locked Bag 2001 Kirrawee DC NSW 2232 Australia; ^6^ School of Chemistry The University of Sydney Sydney NSW 2006 Australia; ^7^ Beijing National Laboratory for Condensed Matter Physics Institute of Physics Chinese Academy of Sciences Beijing 100190 China; ^8^ Collaborative Innovation Center of Quantum Matter Beijing 100190 China; ^9^ School of Physical Sciences University of Chinese Academy of Sciences Beijing 100190 China

**Keywords:** cathode materials, composite materials, layered materials, Li/TMs interdiffusion, oxygen redox reaction

## Abstract

To meet the growing demand for global electrical energy storage, high‐energy‐density electrode materials are required for Li‐ion batteries. To overcome the limit of the theoretical energy density in conventional electrode materials based solely on the transition metal redox reaction, the oxygen redox reaction in electrode materials has become an essential component because it can further increase the energy density by providing additional available electrons. However, the increase in the contribution of the oxygen redox reaction in a material is still limited due to the lack of understanding its controlled parameters. Here, it is first proposed that Li‐transition metals (TMs) inter‐diffusion between the phases in Li‐rich materials can be a key parameter for controlling the oxygen redox reaction in Li‐rich materials. The resulting Li‐rich materials can achieve fully exploited oxygen redox reaction and thereby can deliver the highest reversible capacity leading to the highest energy density, ≈1100 Wh kg^−1^ among Co‐free Li‐rich materials. The strategy of controlling Li/transition metals (TMs) inter‐diffusion between the phases in Li‐rich materials will provide feasible way for further achieving high‐energy‐density electrode materials via enhancing the oxygen redox reaction for high‐performance Li‐ion batteries.

## Graphical abstract

A new key parameter for fully exploiting the oxygen redox reaction by Li/transition metals (TMs) inter‐diffusion between the two phases in Co‐free Li‐rich materials is first proposed. The resulting Li‐rich materials can achieve fully exploited oxygen redox reaction and thereby can deliver the highest reversible capacity leading to the highest energy density, ≈1100 Wh kg^−1^ among Co‐free Li‐rich materials.

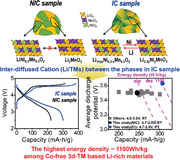

## 1 Introduction

Rechargeable Li‐ion batteries have become a key enabler for transformational changes in our society by powering advanced portable electronics and deploying electric vehicles and grid‐scale applications.^[^ ^1^ ^]^ To meet the soaring demand in high‐energy‐density Li‐ion batteries, an oxygen redox reaction in electrode material has been considered as an essential component because it can provide additional available electrons to overcome the limit of theoretical energy density in conventional electrode materials based only on cationic redox reactions. To realize this transformation, Li‐rich layered materials (Li_1+_ *_x_*TM_1−_ *_x_*O_2_) have recently become one of the most attractive electrode materials because they can exploit the oxygen redox reaction in addition to the transition metal (TM) redox reaction and thereby can deliver much higher capacities (>250 mAh g^−1^) than conventional layered materials (e.g., LiCoO_2_).^[^ ^2^ ^]^

To further exploit the potential of the oxygen redox reaction, typical approach in Li‐rich materials based on 3d‐TMs is the doping with the cobalt, which can improve and stabilize the oxygen redox reaction.^[^ ^3^ ^]^ Several approaches also have been suggested including applying surface structural modification or coatings,^[^ ^4^ ^]^ which can achieve good capacity retention and mitigated voltage fade because surface treatments can suppress surface changes caused by the oxygen gas release on the surface due to irreversible oxygen redox reaction.^[^ ^5^ ^]^ And the other approaches have been focused on replacing 3d‐TMs with 4d or 5d‐TMs^[^ ^2^, ^6^ ^]^ in Li‐rich materials, which can stabilize the reversibility of the oxygen redox reaction by using relatively strong covalent character of 4d‐ or 5d‐ TMs with oxygen compared to 3d‐TMs or by forming a dimer‐like oxygen rather than a unbonded oxygen.^[^ ^7^ ^]^ To achieve high energy density in Li‐rich materials, several different approaches based on disordered‐rocksalt structure have been also proposed recently.^[^ ^6^, ^8^ ^]^ Disordered‐rocksalt Li‐excess materials can have stable framework, which can increase usable Li amount, and achieve reasonable electrochemical activity because of reasonable Li diffusion activated by excess Li. The replacement of oxygen with fluorine in the disordered‐rocksalt Li‐excess materials have been developed to improve electrochemical performance of the materials by suppressing oxygen gas loss^[^ ^5b^ ^]^ or by increasing the contribution of the transition metal redox reaction.^[^ ^8^ ^]^ Most of these previous approaches have focused on stabilizing the oxygen redox reaction and its reversibility or understanding the oxygen redox reaction with 4d/5d‐TMs, rather than the increase in the contribution of the oxygen redox reaction. In order to further increase the energy density, the oxygen redox reaction should be fully exploited by understanding a key parameter that can control/enhance the oxygen redox reaction in Li‐rich materials.

Here, we first report on a novel way of controlling the oxygen redox reaction in Li‐rich materials by using the inter‐diffusion of Li/TMs between the two phases such as rhombohedral and monoclinic phase. As a result, the inter‐diffusion in the Li‐rich materials will increase the cation disordering in both phases and at the same time achieve excess Li in both phases. These structural/chemical changes can fully exploit the oxygen redox reaction in the bulk, enabling full extraction of Li with high reversible capacity, because the excess Li in both phases can provide additional available electrons leading to the increase in the oxygen redox reaction, and significantly improve intrinsic layered structure stability due to the increased cation disordering in both phases in a pristine material, which can improve reversibility of electrochemical reactions simultaneously. The resulting materials achieve reversible capacity of ≈300 mA h g^−1^ even in narrow cut‐off voltage window from 2.5 to 4.7 V with a high operating voltage ≈3.6 V providing the highest energy density of ≈1100 Wh kg^−1^ among reported Co‐free Li‐rich layered materials. Moreover, the inter‐diffused Li‐rich materials can achieve mitigated voltage fade and improved capacity retention for 100 cycles, and can deliver substantially enhanced power capability among other reported Co‐free 3d‐TMs Li‐rich materials. Controlling the oxygen redox reaction via the Li/TMs distribution between the phases in Li‐rich materials can provide a promising opportunity to design high‐energy‐density Li‐ion electrode materials that can achieve highly exploited oxygen redox reaction.

## 2 Results

### 2.1 Composite Structure Characterizations of the Samples

**Figure 1:**
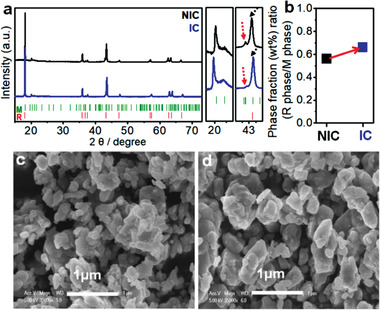
a) Synchrotron X‐ray diffraction (SXRD) of IC and NIC sample (green line: the peaks of the M phase, red line: the peaks of the R phase). b) Phase fraction (wt%) ratio (R phase/M phase) from Rietveld refinements of neutron diffraction (Table S2, Supporting Information) (closed square: phase fraction of R phase, open square: phase fraction of in M phase). c,d) SEM image of NIC sample (c) and IC sample (d).

To understand the oxygen redox reaction, we chose a well‐known Li_1.2_Ni_0.2_Mn_0.6_O_2_ layered material as a model system because it is free of cobalt and has a certain contribution to the obtained capacity by the oxygen redox reaction.^[^ ^9^ ^]^ The samples were prepared by using intimate mixing with a high energy ball‐milling followed by different cooling rates at high temperature (detailed procedures in Figure S1, Supporting Information). Synchrotron X‐ray diffraction (SXRD) (**Figure** **1**a; Figure S2c,d, Supporting Information) and neutron powder diffraction (NPD) (Figure S2a,b, Supporting Information) clearly show that the samples are composed of the two phases, a rhombohedral phase (R‐3m symmetry, “R phase”) such as LiNi_0.5_Mn_0.5_O_2_ (LNMO) and a monoclinic phase (C2/m symmetry, “M phase”) such as Li_2_MnO_3_, which is indicated by the broad superstructure peaks at 20–23° in the 2*θ* range^[^ ^10^ ^]^ (Figure 1a), which is consistent result with STEM image in Figure S3, Supporting Information. Even though both samples form a composite and have the same nominal composition (Table S1, Supporting Information), the samples show different composite structure indicated by different degree of the merge of the peaks between the (20–2) peak of the M phase (red arrow in Figure 1a)^[^ ^10a,b^ ^]^ and the combined peaks of both M and R phase (black arrow in Figure 1a).^[^ ^10a,b^ ^]^ This indicates that the samples can have different interaction between the two phases in a composite. Based on this different interaction in the composite structure, the samples were labelled as an inter‐diffused cation (IC) sample that has a certain degree of interaction between the two phases and Not‐IC (NIC) sample that does not have any interaction. The IC sample shows that the (20–2) peak of the M phase are merged with the combined peaks of the both M and R phase, whereas the NIC sample shows the separation of these peaks in Figure 1a.

To figure out the interaction between the two phases, NPD and SXRD measurements were carried out in the samples (Figure S2 and Tables S2 and S3, Supporting Information). Refinement results of NPD in Figure 1b show that the two samples have quite different phase fraction of the R phase (such as LNMO phase) and the M phase (such as Li_2_MnO_3_ phase) in the composite even though they have the same nominal composition of Li_1.2_Ni_0.2_Mn_0.6_O_2_ (Table S1, Supporting Information). The IC sample has higher R phase than the NIC sample but lower M phase than the NIC sample. As a result, the phase fraction ratio (R phase/M phase) increases from 0.562 in the NIC sample to 0.666 in the IC sample. This indicates that the IC sample has the interaction between the two phases, such as cation inter‐diffusion between the two phases, and thereby can have different chemical composition compared to the NIC sample considering that the two samples have the same nominal composition. It should be noted that the R phase in the samples can be easily decomposed to the M phase with Ni‐based layered material at high temperature leading to slightly higher fraction of the M phase in the samples compared to the nominal composition.^[^ ^11^ ^]^ Given the merge of peaks between the two phases in the IC sample in Figure 1a, the IC sample can possibly have higher cations inter‐diffusion between the two phases. It should be noted that the two samples have similar particle size, >200 nm and morphology (Figure 1c,d) indicating that the interaction between the two phases can happen in a particle (detailed in Figure S3, Supporting Information).

### 2.2 Chemical and Local Structure Characterizations of the Samples

**Figure 2:**
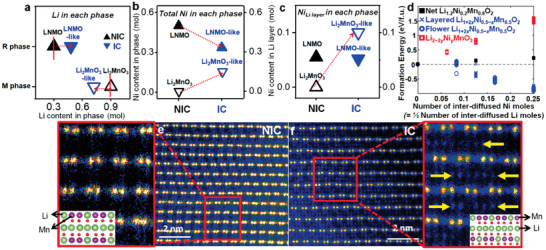
a) The amount of Li in the two layered environments based on deconvoluted lines obtained from ^6^Li MAS NMR at spinning speeds of 30 kHz (Figure S4 and Table S4, Supporting Information) (closed: R phase environment, open: M phase environment). Site occupancies of the amount of Ni atoms in each phase, as determined by Rietveld refinements of Neutron diffraction. b) The amount of total Ni atoms in each phase (related with the amount of diffused Ni atoms from R phase to M phase. c) The amount of Ni atoms in Li layer (the degree of cation disordering) of each phase (closed: R phase environment, open: the M phase environment). d) DFT calculations of net formation energies of the materials as a function of the amount of diffused Ni into Li_2_MnO_3_ phase or the 1/2 amount of diffused Li into LNMO phase for Li‐Ni inter‐diffusion of each layered phase at *T* = 0 K. HAADF‐STEM image of the M phase in the e) NIC sample projected along the [100]_M_ direction, f) IC sample projected along the [1–10]_M_ and [110]_M_ direction (Figure S5, Supporting Information) (inset: atomic configuration in a unit cell of M phase [such as Li_2_MnO_3_], green: Li and purple: Mn).

To understand how the inter‐diffused cations between the two phases affects local Li environments, ^6^Li magic‐angle‐spinning (MAS) nuclear magnetic resonance (NMR) measurements were carried out in the samples (detailed in Figure S4 and Table S4, Supporting Information). Four main different local environments of Li were distinguished:^[^ ^12^ ^]^ Li in Li layers or TM layers in each R phase and M phase. The amount of Li (mol) in the R phase environment (**Figure** **2**a) increased from 0.30 mol in the NIC sample to 0.50 mol in the IC while the amount of Li in the M phase environment decreased from 0.90 mol in the NIC sample to 0.70 mol in the IC sample. Given that the phase fraction of R/M phase in the IC sample in Figure 1b is similar to that of the nominal composition (Li_2_MnO_3_–LiNi_0.5_Mn_0.5_O_2_), Li amount (≈0.5 mol) of the R phase (Figure 2a) in the IC sample is higher than that (≈0.4 mol) of the R phase in the nominal composition while Li amount (≈0.9 mol) of the M phase is lower than that (≈0.8 mol) in the nominal composition. This indicates that some of Li can be inter‐diffused into the R phase from the M phase leading to the formation of the LNMO‐like phase and Li_2_MnO_3_‐like phase. It should be noted that the NIC sample has different amount of Li in the R/M phase compared to the nominal composition due to different phase fraction rather than other interactions.

As a result, the IC sample has higher amount of Li in LNMO phase but lower amount of Li in Li_2_MnO_3_ phase compared to the nominal composition. The inter‐diffused Li between the two phases can lead to excess Li in LNMO phase in addition to excess Li in Li_2_MnO_3_ phase but the NIC sample does not have this (Figure 2a). Considering that each phase in a composite should obey a charge neutrality, the inter‐diffused Li between the two phases in the IC sample should be compensated. One of possible compensation mechanism can be the diffusion of transition metals from the R phase to the M phase, the opposite direction of the Li diffusion.

NPD measurements on the samples in Figure 2b (Table S2, Supporting Information) clearly show that the IC sample has a certain amount of diffused TM from the R phase into the M phase. Especially, Ni^2+^ ions from the LNMO phase into the Li_2_MnO_3_ phase (Figure 2b) can be possible rather than Mn^4+^ ions by considering the electrostatic interaction. This Ni inter‐diffusion can lead to the incorporation of Ni in Li_2_MnO_3_ phase in addition to the excess Li in LNMO phase in the IC sample. The total amount of diffused Ni obtained from refinements of the neutron data is ≈0.14 mol between the two phases in the IC sample. Some of diffused Ni (≈0.10 mol) into the M phase in the IC sample can be incorporated into Li layers (Figure 2c) leading to the increase in the cation disordering (Ni in Li layers) whereas the NIC sample does not have any Ni incorporation in the M phase, even though the amount of Ni in Li layers of the R phase is similar for both samples. These NMR and neutron results clearly indicate that the two phases in the IC sample have different degree of cation disordering and different compositions with respect to Li and Ni compared to those phases in the NIC sample and in the nominal composition. In other words, the IC sample is composed of the composite of Ni incorporated Li_2_MnO_3_ (Li_2_MnO_3_‐like) phase with the increased cation disordering and excess Li LNMO (LNMO‐like) phase but the NIC sample does not have these features of the two phases.

Furthermore, density functional theory (DFT) calculations clearly demonstrate our observations in the IC sample (details about DFT calculation in Supporting Information). We modeled the two phases after Li/Ni inter‐diffusion, which are initially predicted based on the electrostatic interaction energies; Li_2−2_ *_y_*Ni*_y_*MnO_3_ and the Li_1+2_ *_x_*Ni_0.5−_ *_x_*Mn_0.5_O_2_. To model the Li_1+2_ *_x_*Ni_0.5−_ *_x_*Mn_0.5_O_2_ phase, the layered and flower structures were used as a starting structure. The flower structure of the Li_1+2_ *_x_*Ni_0.5−_ *_x_*Mn_0.5_O_2_ phase is from one of honeycomb Ni/Mn arrangements with Li disordering in TM layer in the layered structure.^[^ ^13^ ^]^ The net formation energies of the composite that has certain amount of Li‐Ni inter‐diffusion between LNMO and Li_2_MnO_3_ increase linearly but are slightly positive as the amount of inter‐diffused Li/Ni (*x*) increases in Figure 2d. Since high thermal energy can be provided by experimental conditions and configurational entropies can be increased by inter‐diffused cations between the two phases, this slightly positive net formation energies can be easily overcome at high temperature leading to the formation of the composite, which have the two layered phases with LNMO‐like phase, which has excess Li in interstitial sites and less Ni compared to LiNi_0.5_Mn_0.5_O_2_ (Figure S4 and Table S2, Supporting Information), and Li_2_MnO_3_‐like phase, which has Ni incorporation and less Li compared to Li_2_MnO_3_ phase.

Further evidence of the Ni incorporation into the Li_2_MnO_3_‐like phase in the IC sample is provided by high‐angle annular dark‐field scanning transmission electron microscopy (HAADF‐STEM) images (Figure 2e,f). It should be noted that HAADF mode can only detect heavy elements such as Ni or Mn. HAADF image of the Li_2_MnO_3_ phase in Figure 2e clearly no additional atoms in Li layers in Li_2_MnO_3_ phase projected along [100]_M_ direction, which is general feature of Li_2_MnO_3_ phase.^[^ ^14^ ^]^ However, HAADF image of the Li_2_MnO_3_‐like phase projected along [1–10]_M_, [110]_M_ direction in Figure 2f clearly shows lots of Ni atoms in Li layers (yellow arrow). This is consistent with the Ni occupancy in the Li_2_MnO_3_‐like phase (Table S2, Supporting Information). This indicates that the IC sample has the Ni incorporation into the Li_2_MnO_3_‐like phase and can have the increased cation disordering in Li_2_MnO_3_ phase.

In order to further confirm the change of the transition metals in the local structure of the samples, the temperature‐dependent magnetic susceptibility measurements (Figure S6, Supporting Information) were carried out. It clearly demonstrates that the IC sample has lower the magnetic ordering from LiMn_6_‐like region (LiMn_6_ ordering) of the M phase^[^ ^15^ ^]^ than the NIC sample. Given that the magnetic ordering in the M phase is from Mn arrangements, lowering magnetic ordering indicates that the M phase in the IC sample can have a changed local structure by Li‐Ni inter‐diffusion with the increased cation disordering (detailed explanation in Supporting Information). In addition, extended X‐ray absorption fine structure (EXAFS) spectra (Figure S7, Supporting Information) also clearly show that a higher degree of local structural disorder in the IC sample than NIC sample was obtained by comparing structural parameter such as the Debye–Waller factor^[^ ^14^, ^16^ ^]^ (detailed explained it in Supporting Information).

Neutron, NMR, STEM data, and DFT calculations in Figures 1 and 2 suggest that the IC sample has the Li‐Ni inter‐diffusion between the two phases, whereas the NIC sample does not have this interaction. The most possible way of the inter‐diffusion between the two layered phases in the IC sample is as follows; 2Li^+^ ions from the Li_2_MnO_3_ phase can diffuse to the LNMO phase and simultaneously Ni^2+^ ions from the LNMO phase can diffuse to the Li_2_MnO_3_ phase for satisfying a charge balance without any changes in the Ni/Mn oxidation states (Figure S8, Supporting Information). Therefore, the Li‐Ni inter‐diffusion in the IC sample leads to the excess Li in the R phase and Ni incorporation in the M phase with the increased cation disordering and thereby can cause different compositions of the two phases from those in the NIC sample even though overall composition of the IC sample is the same as the NIC sample.

### 2.3 Electrochemical Properties and Oxygen Redox Reaction in the Samples during Charge/Discharge at RT

**Figure 3:**
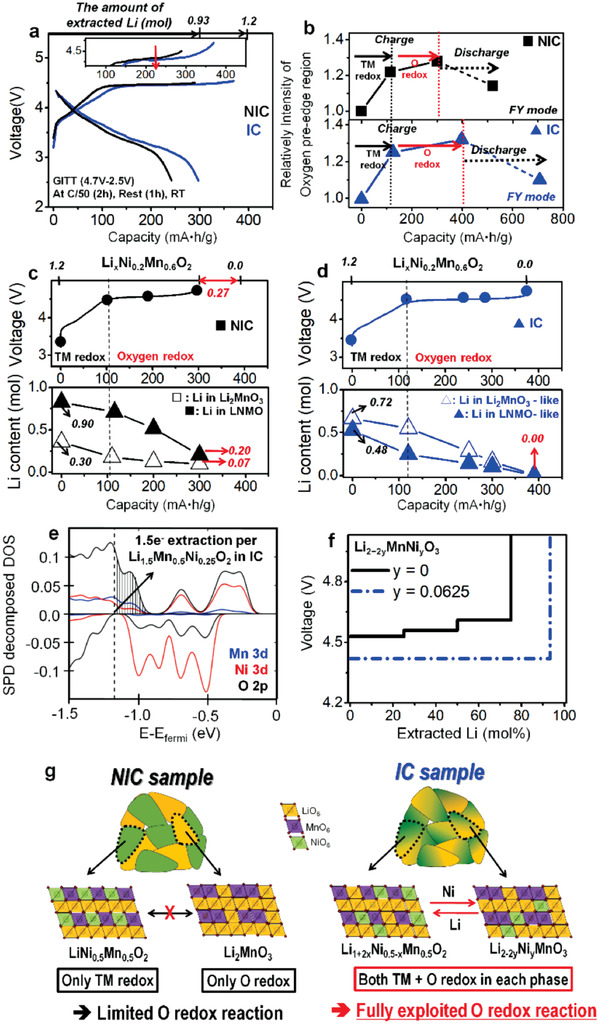
a) Open circuit voltage (OCV) curves of the samples at 2.5–4.7 V and C/50 (5.6 mA g^−1^) at RT (from galvanostatic intermittent titration technique [GITT]). b) Variation of integrated intensity in the low‐energy region (<534 eV) of the oxygen K edge during first cycle in fluorescence yield (FY, bulk sensitive) mode for both IC sample and NIC sample by ex situ SXAS measurements of electrodes during first cycle. Voltage profiles of first charge at C/20 rate (14 mA g^−1^) in 2.5–4.7 V. The amounts of Li in the two layered phases estimated by ex situ NMR measurements of the electrodes at different states of charge (SOCs) for c) NIC sample (closed square: LNMO phase, open square: Li_2_MnO_3_ phase) and d) IC sample (closed triangle: LNMO‐like phase, open triangle: Li_2_MnO_3_‐like phase). e) Projected density of states of Li_1.5_Ni_0.25_Mn_0.5_O_2_ (i.e., Li‐excess LNMO phase in the IC sample via 25% Ni diffused to Li_2_MnO_3_ phase and 50% Li excess in LNMO phase). f) Calculated redox potential during Li extraction of the Li_2−2_ *_y_*MnNi*_y_*O_3_ (Li_2_MnO_3_‐like phase) as the amount of Li‐Ni inter‐diffusion (*y* = 0 and 0.0625) between the two layered phases. g) Schematic diagrams of possible atomic structure at local scales and redox reactions in the samples.

The Li‐Ni inter‐diffusion between the two phases in the IC sample significantly improves electrochemical activities in **Figure** **3**a. The IC sample achieves almost full charge capacity, ≈390 mAh g^−1^ indicating full extraction of Li, which is further confirmed by the negligible (≈0.002 mol) residual Li in a fully charged electrode with ICP (inductive‐coupled plasma) method, whereas the NIC sample can achieve only ≈300 mAh g^−1^, that is only ≈0.93 mol Li extraction. Even though almost all Li ions are extracted from the layered structure in first charge process in the IC sample, the IC sample delivers remarkably higher discharge capacity, ≈300 mAh g^−1^ and higher discharge voltage of ≈3.6 V than the NIC sample in Figure 3a. The initial columbic efficiency of the two samples is similar with each other; 83% for the NIC sample and 81% for the IC sample. Even though the IC sample has much longer voltage plateau at ≈4.5 V than the NIC sample, similar initial coulombic efficiency of the IC sample with the NIC sample means that the IC sample has substantially improved oxygen redox reversibility rather than large irreversible oxygen redox reaction such as the oxygen loss at initial cycle.^[^ ^2b,g^ ^]^ This indicates that the Li‐Ni inter‐diffusion in the IC sample can improve the layered structure stability leading to a robust structure. The full Li extraction (≈1.2 mol) with long plateau at ≈4.5 V and high reversible discharge capacity in the IC sample (Figure 3a) has been first reported in this kind of Co‐free Li‐rich materials. This indicates that Li‐TMs inter‐diffusion between the two layered phases in Li‐rich materials can enable full Li extraction in Li‐rich materials with high reversible capacity at RT.

To understand the oxygen redox reaction in the samples, X‐ray absorption spectroscopy (XAS) measurements were carried out. The Ni K‐edge XANES data during first cycle (Figure S9, Supporting Information) shows that the Ni redox reaction from 2+ to 4+ in the samples can contribute up to ≈125 mAh g^−1^ to the obtained capacity. This is consistent with previous results in the literature.^[^ ^2b,g^ ^]^ As a result, the oxygen redox reaction in the IC sample can contribute the additional capacity, ≈175 mAh g^−1^ to the obtained discharge capacity indicating substantial improvement of reversible oxygen redox contribution compared to the NIC sample, ≈125mAh g^−1^ from the oxygen redox reaction (Figure 3a). Improved oxygen redox reaction in the IC sample is clearly supported by the integrated intensity of the pre‐edge region below ≈534 eV in the O K‐edge SXAS (soft X‐ray absorption spectroscopy) spectra (Figure S10 and Table S6, Supporting Information). This region can be attributed to transitions from the O 1s to the unoccupied state in O 2p orbital, and to transitions from the O 1s to the empty hybridized TM d—O 2p orbitals.^[^ ^2^, ^17^ ^]^ Given that the changes in the oxidation state of the Ni and Mn is negligible above 125 mAh g^−1^ (Figure S9, Supporting Information), the changes in the integrated intensity of the pre‐edge region at <534 eV in the O K‐edge in the samples indicate that the oxygen redox reaction can be contribute for achieving additional capacity.^[^ ^9^, ^18^ ^]^ Concomitantly, the IC sample shows much higher increase in the integrated intensities of the O K‐edge in both bulk (FY mode, in Figure 3b) and surface regions (TEY mode, in Figure S10, Supporting Information) during first cycle than the NIC sample. These results support that the IC sample has the increase in the oxygen redox contribution and reversibility compared to the NIC sample.

To understand the extraction of Li and the activated oxygen redox reaction in the samples, NMR measurements on the ex situ electrodes during first charge process were carried out. Figure 3c (Figure S11, Supporting Information) shows that the NIC sample cannot achieve full Li extraction indicated by the obtained charge capacity (≈300 mAh g^−1^); the LNMO phase and Li_2_MnO_3_ phase in the NIC sample is not highly activated, leaving ≈0.07 mol Li and ≈0.20 mol Li in the structure at the end of charge, respectively (Figure 3c; Figure S11, Supporting Information). This limited electrochemical reaction in the NIC sample can be related to much shorter voltage plateau at ≈4.5 V, where the oxygen redox reaction mainly occurs^[^ ^2d,e^ ^]^ in Figure 3c. NMR data in the NIC sample clearly shows that during the voltage plateau at ≈4.5 V, the LNMO phase shows a negligible additional delithiation and at the same time the Li_2_MnO_3_ phase shows a limited delithiation. This indicates that the NIC sample has a limited oxygen redox reaction. In contrast, Figure 3d (Figure S12, Supporting Information) shows that the charge capacity of ≈390 mAh g^−1^ in the IC sample was mainly from the full extraction of Li caused by the redox reactions rather than the electrolyte decomposition or other side reactions at high voltage. The full charge capacity in the IC sample is due to the long voltage plateau at ≈4.5 V, where the oxygen redox reaction mainly occurs.^[^ ^2d,e^ ^]^ NMR data in Figure 3d clearly shows that the long voltage plateau at ≈4.5 V in the IC sample is ascribed to an unexpectedly large amount of Li extracted from the LNMO‐like environments (≈0.25 mol Li extraction during 4.5 V plateau in Figure 3d) in addition to full Li extraction from the Li_2_MnO_3_‐like environments (≈0.55 mol Li extraction during 4.5 V plateau in Figure 3d) without leaving any residual Li. Considering that further oxidation of Ni^4+^/Mn^4+^ ions is not available (Figures S9 and S10, Supporting Information) above ≈4.5 V, this unexpected full Li extraction from both the LNMO‐like and Li_2_MnO_3_‐like environment in the IC sample means that the oxygen redox reaction is almost fully exploited. Considering that the IC sample has full charge capacity in both the LNMO‐like phase and Li_2_MnO_3_‐like phase but the NIC sample does not, fully exploited oxygen redox reaction in the IC sample can be ascribed to different composite structure caused by the inter‐diffused cations between the two phases in Li‐rich materials.

The Li‐Ni inter‐diffusion between the two phases in the IC sample can fully exploited oxygen redox reaction in Li‐rich materials allowing full extraction of Li with high reversible discharge capacity of ≈300 mAh g^−1^ and a high discharge potential, ≈3.6 V. Thus, the IC sample can achieve ≈1100 Wh kg^−1^, which is the highest energy density compared to reported Co‐free Li‐rich materials (in Figure S14, Supporting Information). To understand the effects of fully exploited oxygen redox reaction on the electrochemical properties of the IC sample, DFT calculations were carried out in both LNMO‐like phase and Li_2_MnO_3_‐like phase. Predicted electronic density of states (DOS) (Figure 3e; Figure S13, Supporting Information) show that the excess Li in the LNMO‐like phase can increase additional electronic states nearby the O 2p orbital. These additional states can provide additional available electrons for the oxygen redox reaction (Figure 3e) when sufficient Li ions are extracted. This indicates that the LNMO‐like phase in the IC sample can have the activated oxygen redox reaction leading to the increased achievable capacity. This result is consistent with the ex situ NMR results (Figure 3d), which shows full Li extraction in the LNMO‐like phase in the IC sample. In contrast, DOS (density of state) calculations in the NIC sample indicate that the LNMO phase does not have any available additional electronic states (Figure S13a, Supporting Information) partly due to the absence of excess Li in it. In addition, DFT calculations clearly show that the Ni incorporation into Li_2_MnO_3_ phase like Li_2_MnO_3_‐like phase in the IC sample can help to improve its electrochemical activity and decrease the redox potential during Li extraction (Figure 3f) compared to the Li_2_MnO_3_ phase. For example, the Li_1.875_Ni_0.0625_MnO_3_ (blue dash line in Figure 3f) can achieve almost full Li extraction below 4.7 V with almost fully exploited oxygen redox reaction. This indicates that the oxygen redox reaction in the Li_2_MnO_3_‐like phase in the IC sample can be fully exploited leading to full extraction of Li as shown in NMR result. In contrast, the Li_2_MnO_3_ phase without any Ni incorporation (black solid line in Figure 3f) cannot achieve full Li extraction below 4.7 V with limited oxygen redox reaction. This indicates that the oxygen redox reaction in the Li_2_MnO_3_ phase in the NIC sample cannot be fully exploited leading to limited extraction of Li. These calculation results are consistent with the NMR results of ex situ electrodes (Figure 3c,d). Furthermore, the redox potential in the Li_1.875_Ni_0.0625_MnO_3_ phase (blue dash line in Figure 3f), which is Ni incorporated Li_2_MnO_3_‐like phase, compared to that in the Li_2_MnO_3_ phase (black solid line in Figure 3f) is lowered, which is consistent with the observation of the voltage plateau at ≈4.5 V in first charge (inset in Figure 3a) in IC sample compared to the NIC sample.

Oxygen K‐edge SXAS, NMR data of ex situ electrodes and DFT calculations in Figure 3 suggest that the IC sample show full Li extraction via fully exploited oxygen redox reaction with Ni redox reaction in both LNMO‐like phase and Li_2_MnO_3_‐like phase, whereas the NIC sample does not show full Li extraction due to limited oxygen redox reaction from the separated redox reactions, TM redox reaction in only LNMO phase and limited oxygen redox reaction in only Li_2_MnO_3_ phase (Figure 3g). Therefore, the Li‐Ni inter‐diffusion between the two phases in Li‐rich materials will be a critical factor in increasing the contribution of the oxygen redox reaction to achievable capacity.

### 2.4 Electrochemical Performance of the Samples at RT

**Figure 4:**
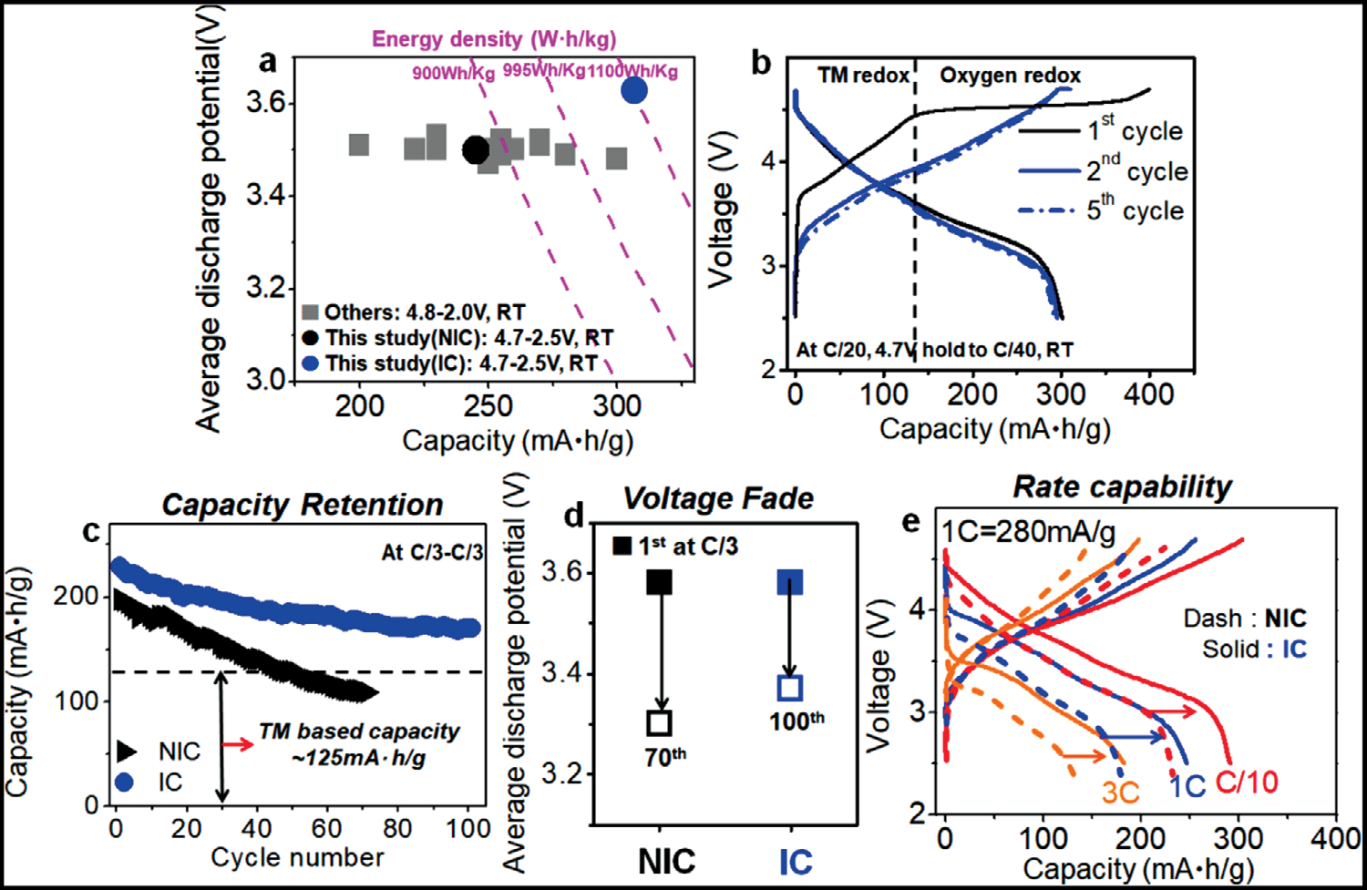
a) Average discharge potential versus discharge capacity with energy density plot among Co‐free Li_1.2_Ni_0.2_Mn_0.6_O_2_ compounds, including the NIC sample and the IC sample. b) Cycling performance of the IC sample at C/20 rate (14 mA g^−1^) for five cycles, first cycle: 4.7–2.5 V, after first cycle: 4.7 V hold to 4 mA g^−1^. c) Capacity retention of the IC sample and NIC sample for cycles. d) Changes in average discharge potential at C/3 charge and C/3 discharge rates for cycles (Figure S15, Supporting Information). e) Rate capability of the IC sample (solid line) and NIC sample (dash line) sample. All electrochemical tests were performed at room temperature and the operating voltage window was from 2.5 to 4.7 V. And average discharge potential obtained at half discharge capacity or half energy density during discharge.

Extraordinary improvement of oxygen redox activity achieved by the Li‐TMs inter‐diffusion between the two phases in the Li‐rich materials can provide additional electrons to obtain more capacity. The IC sample in a relatively narrow 4.7–2.5 V cut‐off voltage window compared to other reported data can achieve the highest discharge capacity of ≈305 mAh g^−1^ via enhancing reversible oxygen redox reaction with a high discharge potential, ≈3.62 V. It can deliver the highest energy density, ≈1100 Wh kg^−1^ among reported Co‐free Li‐rich materials (**Figure** **4**a; Figure S14, Supporting Information). Furthermore, the IC sample shows high reversible discharge capacities (>300 mAh g^−1^) even in subsequent cycles at low rate (C/20) with a high discharge voltage of ≈3.6 V (Figure 4b) even after full extraction of Li during the first charge process (≈390 mAh g^−1^). Based on the TM redox reaction ≈125 mAh g^−1^, significantly enhanced oxygen redox reaction in subsequent cycles (Figure 4b) indicates that the IC sample has stable reversible oxygen redox reaction with a robust composite structure induced by inter‐diffused cations between the phases allowing to reversibly extract/insert large amount of Li. Therefore, excess Li with the increased cation disordering in each phase induced by inter‐diffused Li‐TMs between the two layered phases significantly enhances the oxygen redox activity and improves its reversibility, and thereby yields high reversible capacity for cycles.

The IC sample also shows remarkably improved capacity retention and mitigated voltage fade with cycling (Figure 4c,d). However, the NIC sample showed rapid capacity fade from ≈200 to ≈100 mAh g^−1^, and poor voltage retention with ≈4.3 mV per cycle fading rate during 70 cycles. The IC sample achieved particularly superior (≈85%) capacity retention with only ≈0.6 mV per cycle fading rate during 100 cycles along with improved voltage retention (Figure S15, Supporting Information). This extended cycle stability indicates that the redox reactions such as the oxygen redox and TM‐based redox reaction^[^ ^2a,b^ ^]^ in Li‐rich layered materials can be reversible and sustainable by controlling composite structure via Li‐TMs inter‐diffusion between the two phases. The degree of cation disordering (Ni ions in Li layers) in each phase in the pristine material (Figure 2c; Table S2, Supporting Information) can affect the capacity retention and mitigated voltage fade in the Li‐rich materials. Considering that the IC sample has higher the degree of the cation disordering, especially in the Li_2_MnO_3_ phase than the NIC sample, this improved capacity retention and mitigated voltage fade in the IC sample indicates that the cation disordering can critically affect electrochemical properties in the Li‐rich layered materials. This can be partly because the cation disordering can improve layered structure stability even with large extraction of Li and prevent the migration of Mn in the structure due to strong electrostatic repulsion of Ni ions in Li layers.^[^ ^19^ ^]^ Therefore, controlling the inter‐diffusion of Li‐TM between the phases in the Li‐rich materials can be one of effective ways to improve the capacity retention and mitigate the voltage fade. Furthermore, the IC sample had much higher rate capability than the NIC sample in Figure 4e. A rate of *n*C corresponds to a full discharge in 1/*n* h. The IC sample can achieve ≈250 mAh g^−1^ of capacity at 1C discharge rate (280 mA g^−1^), ≈200 mAh g^−1^ at 3C discharge rate, whereas the NIC sample shows only ≈170 mAh g^−1^ at 1C discharge rate. Improved rate capability with high discharge capacity in the IC sample indicates that the oxygen redox reactions can be kinetically comparable to TM‐only redox reactions, contrary to previous observation in Li‐rich 4d/5d TM oxides.^[^ ^20^ ^]^ The robust layered structure caused by Li‐Ni inter‐diffusion in the IC sample can help to stabilize the fully delithiated phase leading to less polarization at high rates. This can enable high rate capability of the IC sample.

## 3 Conclusion

Controlled structural/chemical changes induced by the Li‐TM inter‐diffusion between the two phases in the Li‐rich materials can help to fully exploit oxygen redox reaction in addition to TM redox reaction, resulting in very high energy density. Given the Li‐rich layered materials that are composed of the two layered phases,^[^ ^3^, ^10^, ^14^, ^21^ ^]^ the Li‐TM inter‐diffusion between both phases can lead to different composite structure such as the excess Li and Ni incorporation in both layered phases that can remarkably improve reversible oxygen redox activity and layered structure stability. The excess Li in the both layered phases can increase local Li‐rich environments such as Li–O–Li structures leading to the formation of available additional electronic states nearby the oxygen orbital that can activate and increase the oxygen redox reaction.^[^ ^2a^ ^]^ Also, Ni incorporation in both layered phases can increase accessible capacity via tailoring electronic structure for easy charge transfer between oxide ions and TM ions^[^ ^22^ ^]^ or via improving layered structure stability caused by the increase in the cation disordering. Thus, both electronic structure and structural stability should be satisfied to achieve high exploiting oxygen redox reaction, especially 3d‐TM based Li‐rich layered materials without any doping or surface coating. As a result, the strategy of controlling Li‐TMs inter‐diffusion between the phases in a Li‐rich material can allow to fully exploit the reversible oxygen redox reaction resulting in increase in the achievable energy density.

In summary, we provide clear demonstration that the controlled structural/chemical changes induced by the Li‐TM inter‐diffusion between the two phases in Li‐rich composites can be critical factor for highly activating the oxygen redox reaction leading to the increase in reversible energy density. This strategy of controlling Li‐TMs inter‐diffusion between the phases in Li‐rich materials will open new avenues for developing the electrode materials that can exceed the capacity of the conventional TM‐only redox materials by using a highly reversible oxygen redox reaction for high performance Li‐ion batteries. We further suggest that our strategy of fully exploited oxygen redox reaction via Li/TM inter‐diffusion between phases in Li‐rich layered materials can be widely applicable to various energy‐related electrochemical systems such as fuel cells and electrochemical catalysts to control electrochemical oxygen redox reaction via controlled structural/chemical changes induced by cations distribution between the components in a composite.

## 4 Experimental Section

##### Preparation of Materials

Li_1.2_Ni_0.2_Mn_0.6_O_2_ were synthesized by solid‐state reaction in four steps or two steps. 1) Appropriate ratios of Li_2_CO_3_, MnO_2_, and NiCO_3_ were ball‐milled in acetone for 12 h. These samples had the molar ratio of Li:Ni:Mn = 1.2:0.2:0.6. 2) A mix of precursors was pelletized, then calcined at 900 °C for 10 h in air with ramping temperature 5 °C min^−1^. 3) The calcined pellets were grounded and pulverized by planetary wet ball‐milling (PBM, Fritsch Pulverisette planetary ball‐mill) for 3 h with 500 rpm. It should be noted that the samples prepared without undergoing the PBM process could exhibit poor oxygen redox activity due to insufficient interaction between Li_2_MnO_3_ and LiNi_0.5_Mn_0.5_O_2_ at high temperatures despite the quenching process (Details in Supporting Information). The pulverized powder was re‐pelletized. 4) The pellets were reannealed for 5 h in air at 800 °C with ramping temperature 5 °C min^−1^ (sample name: inter‐diffused cation, IC) then quenched to room temperature (RT). Alternatively, the pellet was reannealed at 800 °C for 5 h in air, then cooled naturally to RT (sample name: not inter‐diffused cation, NIC). Detailed synthesis processes are explained in Supporting Information.

##### Material Characterizations

The neutron diffraction measurements were performed at the Australian Centre for Neutron Scattering. The wavelength was 1.5340 Å and the scan range was 10–150° in increments of 0.05. All data were collected at RT. The synchrotron X‐ray diffraction measurements were performed on beamline 9B‐HRPD at Pohang Accelerator Laboratory (PAL), Pohang, Korea. The incident X‐rays were vertically collimated by a mirror, and then monochromated to the wavelength of 1.4970 Å by a double‐crystal Si (111) monochromator. The datasets were collected in the range of 10° ≤ 2*θ* ≤ 130° with a step size of 0.02° (2*θ* range). Rietveld refinements for the lattice parameters of each sample and the quantity of impurities were determined using Full Proof software. High‐resolution transmission electron microscopy (TEM) analysis was conducted on a JOEL JEM‐2200FS microscope fitted with a LaB6 filament at an acceleration voltage of 200 kV.

##### Nuclear Magnetic Resonance Measurements

^6^Li and ^7^Li MAS NMR measurements were performed at RT on a Bruker Avance‐200 spectrometer (B0 = 4.7 T, Larmor frequency *μ*_0_ = 29.45 and 77.78 MHz for ^6^Li and ^7^Li, respectively). The ^6^Li isotope was a spin‐1 nucleus and had the smallest quadrupole moment for any nucleus and it could be considered as a spin‐1/2 nucleus. Therefore, sharp linewidths were achieved with compared to the ^7^Li isotope. In addition, ^6^Li had a much lower gyromagnetic ratio (*γ* ^7^Li/*γ* ^6^Li = 2.6) resulting in a much narrower spinning sideband manifolds and NMR spectra easier to interpret. Thus, despite its lowest sensitivity ^6^Li could be preferred instead of ^7^Li detection. However, in the present study ^7^Li MAS NMR was preferred to analysis cycled samples, because of the small amount of sample recovered from cycled electrodes. MAS spectra were obtained by using a Bruker MAS probe with a cylindrical 2.5 mm (o.d.) zirconia rotor. Spinning speed was varied between 25 and 30 kHz in order to determine the position of the isotropic resonances. ^6^Li and ^7^Li MAS NMR spectra were acquired using an echo (π/2−τ−π−τ) pulse sequence with a *π*/2 pulse of 4.1 µs and 2.3 µs for ^6^Li and ^7^Li, respectively. Recycle time was typically 0.5s. The isotropic shifts, reported in parts per million (ppm), were relative to an external liquid 1 m solution in deionized water of LiCl and ^6^Li enriched LiCl set at 0 ppm.

##### X‐Ray Absorption Spectroscopy Measurements

Hard X‐ray absorption spectra of Mn and Ni K‐edges were collected at the facility installed at beamline 7D at PLS (Pohang Light Source)‐II in transmission mode with the N_2_ gas‐ionization detectors and a Si(111) double‐crystal monochromator detuned to ≈70% of its original intensity to eliminate higher‐order harmonics. The storage ring was operated at 2.5 GeV with an injection current of ≈350 mA. The spectral energies were calibrated by using the first inflection points in the Mn and Ni metal foil spectra as references (i.e., Mn K‐edge = 6539 eV, and Ni K‐edge = 8333 eV). Data pre‐processing operations such as deglitching, energy calibration, normalization, and least square fitting with theory were performed by using IFEFFIT which used the FEFF code. Soft XAS spectra of O K‐edge were recorded at beamline 8A1—SPEM and 10D—XAS KIST of PLS‐II in total electron yield (TEY) mode and fluorescence yield (FY) mode under high vacuum condition with a base pressure of 3 × 10^−10 ^Torr. The spectral energy resolution was 0.1 eV and the monochromator absorption features were normalized by dividing the detected signals (*I*_1_) by the photoemission current, *I*_0_ of a gold mesh placed in the incident beam. All the absorption spectroscopy data were measured at RT. Data pre‐processing operations such as deglitching, energy calibration, normalization, and least square fitting with theory were performed by using WINXAS program with two or three Gaussian functions and one Arc tangent function.

##### Electrochemical Measurements

For electrochemical tests, composite electrodes for IC and NIC sample were made by mixing active material (80 wt%), super‐P carbon (Timcal, 15 wt%), and binder (poly(vinylidene fluoride) (PVDF), 5 wt%) by using a solution of 8% (wt) PVDF in *N*‐methylpyrolidinone. A slurry mixture was tape‐cast on Al foil (Hohsen Corp.) by the doctor blade method. The loading density of the electrode was 2–3 mg cm^−2^. The cells were assembled with Li metal (Hohsen Corp.) in an argon‐filled glove box and tested them on a Maccor 2200 operating in galvanostatic mode using lithium metal as an anode, non‐aqueous electrolyte (1 m LiPF_6_ in ethylene carbonate (EC):diethyl carbonate (DEC) (1:1 by volume, PANAX ETEC Co. Ltd., battery grade), and Celgard 2400 as a separator in a 2032‐coin cell. All cells were tested at room temperature. For ex situ experiments, the device in glovebox was disassembled, taking care not to short‐circuit it. And then the electrodes were rinsed with dimethylcarbonate to remove residual electrolyte salt and allowed them to dry. The electrodes were covered for ex situ study with teflon tape or scotch tape and stored in the glovebox until the experiment was carried out.

##### Density Functional Theory Calculations

Phase stability and voltage profiles of Li_1+2_ *_x_*Mn_0.5_Ni_0.5−_ *_x_*O_2_ and Li_2−2_ *_y_*MnNi*_y_*O_3_ (*x, y* < 1) were predicted based on total energies computed using the Vienna Ab initio Simulation Package (VASP) and the Perdew–Burke–Ernzerhof (PBE) functional in the generalized gradient approximation (GGA) and projector‐augmented wave method. The on‐site interaction was corrected by the Hubbard U parameters of 6.0 and 3.9 eV for the Ni and Mn 3d orbitals, respectively. Spin‐polarized calculations were performed with a plane‐wave energy cutoff = 520 eV and a k‐points grid density with more than 0.01 k‐points per Å^3^. Lattice parameters and atomic positions were optimized until the energies and forces converged to within 10^−5 ^eV per atom and 10^−3 ^eV Å^−1^, respectively. The delithiation voltage profiles of the Li_2−2_ *_y_*MnNi*_y_*O_3_ phases were computed for *x*  =  0 and 0.0625 using 4 and 16 formula units in a computational cell, respectively, and Li/vacancy ordering was tested by sampling 30 configurations with the lowest electrostatic energy at each Li content. For reliable energy band positions in density of states, the Heyd–Scuseria–Ernzerhof (HSE) screened hybrid functional was used instead of the GGA functional. Atomic positions and lattice parameters of the most stable structures predicted from GGA were re‐optimized within HSE, and the pseudopotential, plane‐wave energy cutoff, k‐points grid, and energy and force convergence criteria were set to the same used in the GGA calculations. Details are in Supporting Information.

## Conflict of Interest

The authors declare no conflict of interest.

## Supporting information

Supplementary information
