# Supplementary material for: Fully Exploited Oxygen Redox Reaction by the Inter‐Diffused Cations in Co‐Free Li‐Rich Materials for High Performance Li‐Ion Batteries
Source: Adv Sci (Weinh). 2020 Jul 20;7(17):2001658. doi: 10.1002/advs.202001658 (PMC7507071; doi:10.1002/advs.202001658)
Supplement: Supplementary file 1 — Supplementary information [file ADVS-7-2001658-s001.pdf]

WILEY-VCH

Copyright WILEY-VCH Verlag GmbH & Co. KGaA, 69469 Weinheim, Germany, 2020.

Supporting Information

Fully exploited oxygen redox reaction by the inter-diffused cations in Co-free Li-rich materials for high performance Li-ion batteries

Junghwa Lee, Nicolas Dupre, Mihee Jeong, ShinYoung Kang, Maxim Avdeev, Yue Gong, Lin Gu, Won-Sub Yoon, Byoungwoo Kang**

This file includes:

I. Preparation of materials and their properties

- Detail synthesis process of materials
- Rietveld Refinement details for the pristine materials
- Material properties for the pristine materials
- DFT calculations of formation energies of the IC samples that have the Li-Ni inter-diffusion between the two layered phases

II. Anion redox reaction in both R phase and M phase of the IC sample with full Li extraction.

- The Ni K-edge and Mn K-edge of the IC electrodes during 1cycles using *ex-situ* XANES measurements.
- The Ni K-edge, Mn K-edge and O K-edge of the electrodes during 1cycle using *ex-situ* SXAS measurements.
- The amount of Li of the electrodes with different states of charge (SOCs) using *ex-situ* NMR measurements.
- DFT-predicted density of states in Li-excess LNMO using the Heyd–Scuseria–Ernzerhof (HSE) hybrid functional

III. Electrochemical performance of the samples

IV. References

I. Preparation of materials and their properties

- Detail synthesis process of materials (Not critical effect on only quenching process)

$Li_{1.2}Ni_{0.2}Mn_{0.6}O_2$ was synthesized by a solid-state reaction in four steps. (1) Appropriate ratios of Li_2CO_3, MnO_2, and $NiCO_3$ were ball-milled in acetone for 12 h. These samples had the molar ratio of Li: Ni: Mn = 1.2: 0.2: 0.6. (2) A mix of precursors was pelletized, then calcined at 900 °C for 10 h in air. (3) The calcined pellets were grounded and pulverized by planetary ball-milling (PBM, Fritsch Pulverisette planetary ball-mill) for 3 h (20min milling with 500rpm speed and 10min rest for 9 cycles). The total amount of powder in a jar (45 ml) was approximately 0.5 g with filling approximately 30ml acetone as the solvent, and 1-mm-diameter zirconia balls with approximately 6 g were used as the grinding media. The pulverized powder was re-pelletized. The size of pellet was approximately 10 mm diameter by pressing into 26 MPa pressure (4) The pellets were reannealed for 5 h in air at 800 °C. Then the pellet was quickly dropped on a stainless steel plate and pressed by a stainless steel for quenching. Immediately, the pellet was quickly hammered to powder for much faster cooling down the sample (sample name: Inter-diffused Cation Disordering, **IC**). Alternatively, the pellet was reannealed at 800 °C for 5 h in air, then cooled naturally to RT (sample name: Not Inter-diffused Cation Disordering, **NIC**). To understand the effect of each synthesis process on material's structural and electrochemical properties, we prepared additional samples with modified synthesis process. One is No-PBM + Q sample that has same process with (1)-(2) step but does not have PBM process and then quenching process ; synthesis process (1)-(2)-(4) step expect for (3) step. The other is PBM-only sample that has same process from (1)-(3) but does not have any further heating with slow cooling or quenching process ; synthesis process (1)-(2)-(3) step expect for (4) step.

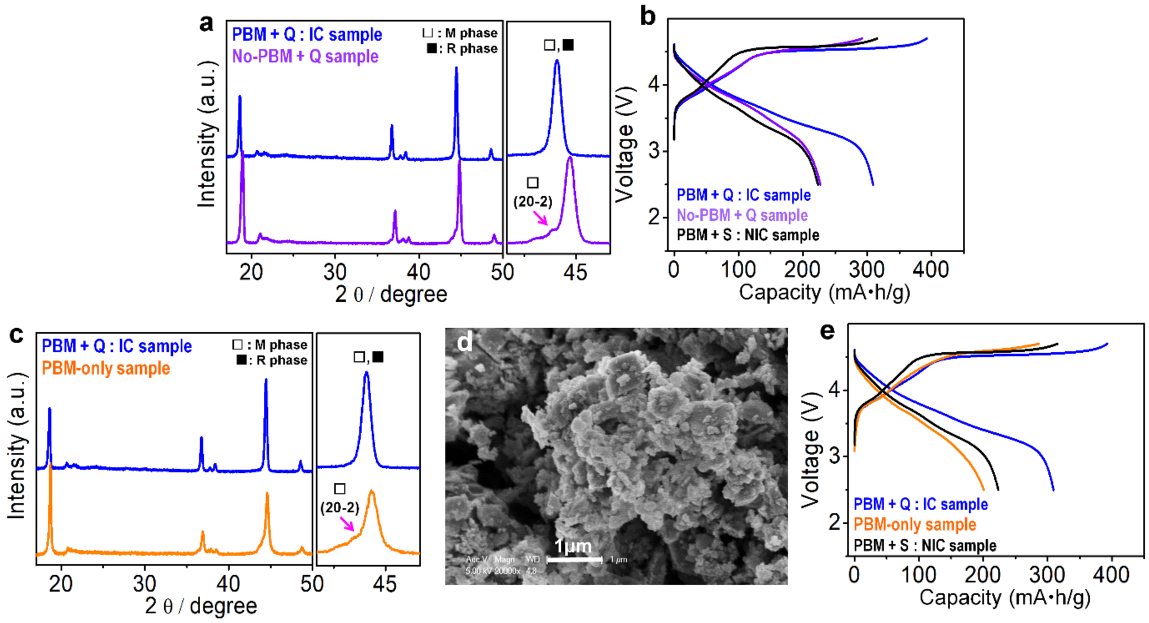

Figure S1. Critical synthesis step of both PBM and quenching process on Li/TMs inter-diffusion between the phases. a, XRD pattern of PBM + Q (IC) sample and No-PBM + Q sample that has the quenching process but does not have PBM process (□: M phase (such as Li_2MnO_3), ■: R phase (such as LNMO)) **b,** Voltage profile of PBM + Q (IC) sample, PBM + S (NIC) sample and No-PBM + Q sample at 2.5 - 4.7V at RT with C/20-C/20 current rate. **c,** XRD pattern of PBM + Q (IC) sample and PBM-only sample without heating process that has only PBM process but does not have any further slow cooling and quenching process (□: M phase (such as Li_2MnO_3), ■: R phase (such as LNMO)) **d,** SEM image of PBM-only sample **e,** Voltage profile of PBM + Q (IC) sample, PBM + S (NIC) sample and Only PBM without heating sample at 2.5 - 4.7V at RT with C/20-C/20 current rate. All samples have the same nominal composition, $Li_{1.2}Ni_{0.2}Mn_{0.6}O_2$.

The No-PBM + Q sample that has the quenching process but does not have any PBM process shows the separation of peaks of R phase (such as $LiNi_{0.5}Mn_{0.5}O_2$) and M phase (Li_2MnO_3) in XRD patterns in Figure S1a. This indicates less interaction between the two phases like the NIC sample (Figure 1a), and thereby shows much poor electrochemical activity than the IC sample that have both the quenching and PBM process in Figure S1b. The No-PBM + Q sample can show poor oxygen redox activity partly due to insufficient interaction between Li_2MnO_3 and $LiNi_{0.5}Mn_{0.5}O_2$ at high temperature even after a quenching process. The result indicates that quenching process by itself is not sufficient for achieving desired Li/TMs inter-diffusion between the two phases. Intimate mixing process via additional high energy

Planetary Ball Milling (PBM) process accompanied with the quenching process is necessary for obtaining desired Li/TMs inter-diffusion in a composite material.

In addition, the PBM-only sample that has only PBM process without any heating and cooling processes in Figure S1c also shows the separation of peaks of R phase and M phase like the NIC sample (Figure 1a). This indicates that the PBM-only sample has less interaction between the two phases, especially Li/TM inter-diffusion between two phases than the IC sample. Even if the PBM-only sample has much smaller particle size (Figure S1d) than the IC sample (Figure 1d), the PBM-only sample shows much poor electrochemical activity, especially oxygen redox activity above 4.5V, than the IC sample (Figure S1e). This indicates that the particle size is not limiting factor for the oxygen redox activity and the Li/TM inter-diffusion between the two phases is not achieved by only PBM process without heating process, which does not have any further slow cooling and quenching process. Thus, both the quenching process and PBM process are needed to achieve certain Li/TMs inter-diffusion between the phases in a composite in Li-rich materials. It should be emphasized that the quenching process accompanied with the PBM process is one of ways to achieve certain Li/TMs inter-diffusion between the phases in a composite.

- **Rietveld Refinement details for the pristine materials**

Rietveld refinements were performed using a two-step strategy.[1]

1. To determine the amount of Ni that had diffused from LNMO phase to Li_2MnO_3 phase, the occupancy of each 3a and 3b site in LNMO phase was constrained regardless of atom species (Li or Ni). Refinement was applied to the first step to determine

   1.1 The amount of deficient Ni compared to total 0.5 mol Ni in LNMO, which will diffuse to Li_2MnO_3 phase, and

   1.2 The amount of Ni/Li exchange between Li layers and TM layers in the R-3m space group, in which only one crystallographic site exists for each layer.

2. The occupancy sites of diffused Ni from the LNMO phase into the Li_2MnO_3 phase and the vacancy sites of Li that can be caused by Li diffusion from the Li_2MnO_3 phase into the LNMO phase sites were refined concurrently.

Refinement results obtained using this strategy were used as a starting crystal structure for neutron powder-diffraction measurements, which can provide more detail information of the structure such as Li occupancy. Even if synchrotron x-ray diffraction refined with this strategy, it is hard to get lot of information than neutron powder diffraction because X-Ray interacts with electron gave a positive intensity but the heavy atom (such as Ni, Mn not Li) dominates the intensity due to its high number of electron unlike neutron. [2]

The amount of Ni that had diffused into Li_2MnO_3 phase and the degree of Li/Ni cation disordering for residual Ni in LNMO phase after Li-Ni inter-diffusion between the two layered phases were also refined from neutron diffraction pattern. In the LNMO ($LiNi_{0.5}Mn_{0.5}O_2$) phase, the IC sample showed not large difference of Li/Ni cation-disordering (~ 0.050 mol) to that of the NIC sample (~ 0.057 mol), which is well-known behavior in the LNMO phase during synthesis.[3] In contrast, in the IC sample, ~ 0.14 mol of Ni had diffused into the Li_2MnO_3 phase from the LNMO phase with increasing cation disordering (Ni occupied the Li layer), whereas in the NIC sample there was no Ni inter-diffusion into the Li_2MnO_3 phase without creating any cation disordering in Li_2MnO_3 phase; these observations indicate that only the LNMO phase in the NIC sample has the cation disordering. In the IC sample, the location of the excess Li in the LNMO phase cannot be specified using these refinements, but Li-NMR results show that excess Li can exist in the LNMO as well as in the Li_2MnO_3 environments. By combined Rietveld refinement of Neutron diffraction patterns and Li-NMR result, IC sample is composed the composite of Li_2MnO_3-like phase and LNMO-like phase,

$0.495Li_{1.72}Ni_{0.14}MnO_3$-$0.505Li_{1.33}Ni_{0.33}Mn_{0.5}O_2$, which is overall composition $Li_{1.2}Ni_{0.2}Mn_{0.6}O_2$, which is same overall composition $0.5Li_2MnO_3$-$0.5LiNi_{0.5}Mn_{0.5}O_2$.

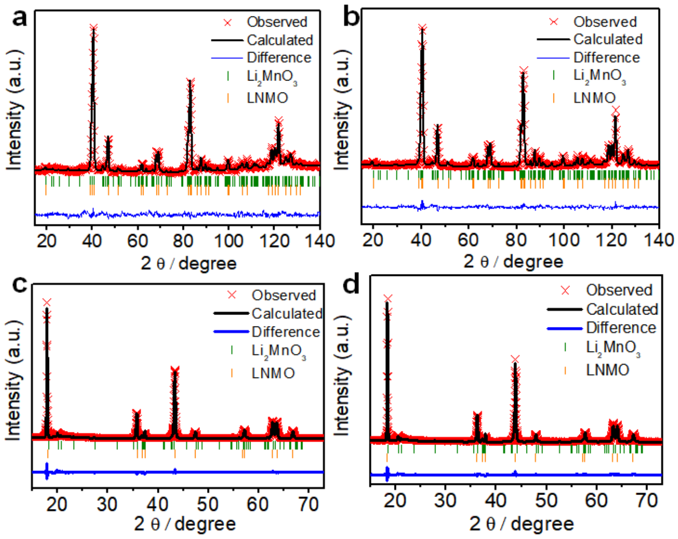

Figure S2. Rietveld refinements of the sample. Neutron powder diffraction (NPD) **a,** NIC sample and **b,** IC sample. Synchrotron X-ray diffraction (SXRD) **c,** NIC sample and **d,** IC sample. Red crosses: observed pattern; black line: calculated diffraction pattern; blue line: difference between observed and calculated patterns. Green and orange vertical lines are theoretical ND patterns of Li_2MnO_3 phase and $LiNi_{0.5}Mn_{0.5}O_2$ phase, respectively. (see Table S2, 3).

- **Material characterizations for the pristine materials**

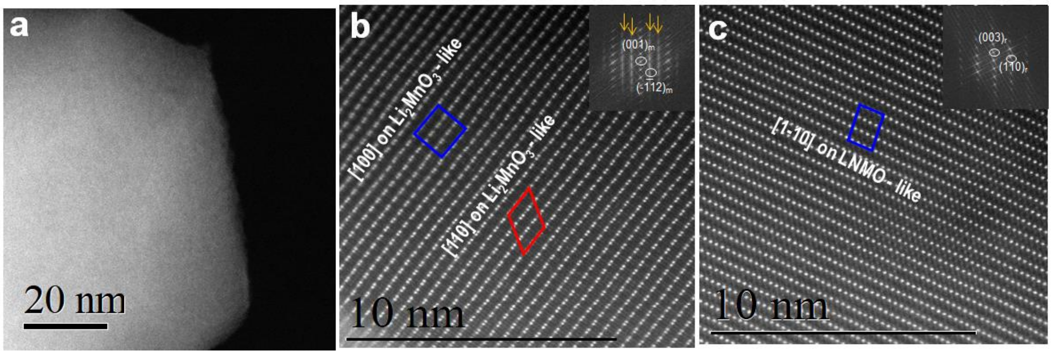

Figure S3. STEM of IC sample. a, STEM image of IC sample **b,** HAADF-STEM image (from Figure S3a) of the monoclinic structure (space group: C/2m, M phase). The spots indicated $(001)_M$ and $(-112)_M$ of the monoclinic phase (donated as M) and elongation of these spots are along variants $[100]_M$, $[1-10]_M$ and $[110]_M$ respectively. **c,** HAADF-STEM image (from Figure S3a) of the rhombohedral structure (space group: R-3m, R phase). The spots indicated with $(003)_R$ of the rhombohedral phase (donated as R) along $[010]_R$.

Given that High-angle Annular Dark Field-Scanning Transmission Electron Microscopy (HAADF-STEM) mode visualizes only heavy atoms such as Ni and Mn,[4] HAADF-STEM images in Figure S3b-c clearly show that the IC sample consists of the two layered phases, the monoclinic phase such as Li_2MnO_3 phase (Figure S3b), which has Li-TM-TM like arrangements, and the rhombohedral phase such as LNMO phase, which has TM-TM-TM like arrangements (Figure S3c).[4] Even though synchrotron XRD data, which can represent the bulk structure, shows the merged peaks in the IC sample in Figure 1a, HAADF-STEM images, which can represent the local structures of a material, clearly show that the two layered phases get separated in domains (Figure S3). This indicates that the IC sample is the composite of the two layered phases, based on TEM images (Figure S3b, c).

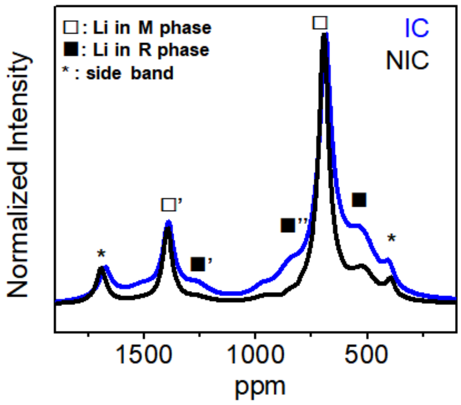

Figure S4. ^{6}Li-NMR data for Li local environments in M phase such as Li_2MnO_3 and R phase such as LNMO in the samples. ^{6}Li-NMR data for Li local environments in M phase such as Li_2MnO_3 and R phase such as LNMO in the samples. Change in local environments of Li in the IC sample was characterized using ^{6}Li MAS NMR measurements (MAS spinning speed of 30 kHz). Four local environments of Li [5] in the samples were distinguished depending on the layer in the phase where the Li atom exists (□: Li in Li layer of M phase, □′: Li in TM layer of M phase, ■: Li in Li layer of R phase , ■′: Li in TM layer of R phase, ■″: Li in interstitial of R phase, *: side bands).

Sharp resonances at ~ 700 ppm (□) and at 1400 ppm (□′) have been assigned to the typical Li local environments found in M phase (such as Li_2MnO_3) environments in Li layers and transition-metal (TM)-layers, respectively. In these environments, Li is surrounded by Mn only. Two broad signals at , ~530 (■), ~1200 ppm (■′) and ~850 ppm (■″) were attributed to Li local environments in R phase (such as LNMO) environments in Li layers and TM layers. The additional broad resonance at 800 - 900 ppm, which is related to Li in the tetrahedral sites (interstitial site) of the spinel structure at ca. 800 - 900 ppm.[6] In those environments, Li is surrounded by both Mn and Ni, in agreement with the lower hyperfine shifts measured compared to their M phase counterparts.[6a] The larger linewidth for Li local environments in R phase than in M phase can be ascribed to a distribution of Li local environments that corresponds to Li with different nearby Ni/Mn ratios and excess Li from octahedral site to tetrahedral site (interstitial site) (Table S4).

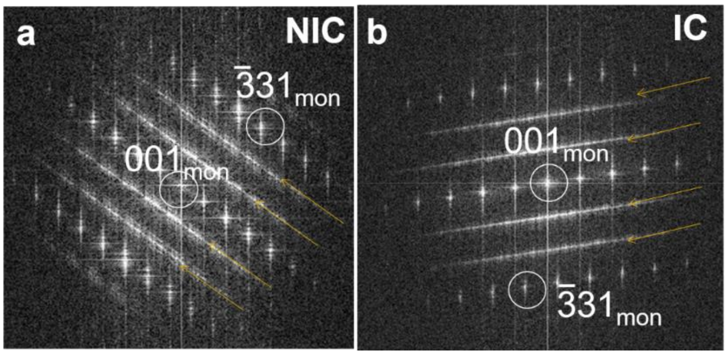

Figure S5. Diffraction pattern of pristine samples. Electron-diffraction (ED) pattern for HAADF-STEM image of the monoclinic structure (space group : C/2m, M phase) in Figure 2e-f. **a,** NIC sample **b,** IC sample. The spots indicated $(001)_M$ and $(-331)_M$ of the monoclinic phase (donated as M) and elongation of these spots are along variants $[100]_M$, $[1\text{-}10]_M$ and $[110]_M$ respectively.

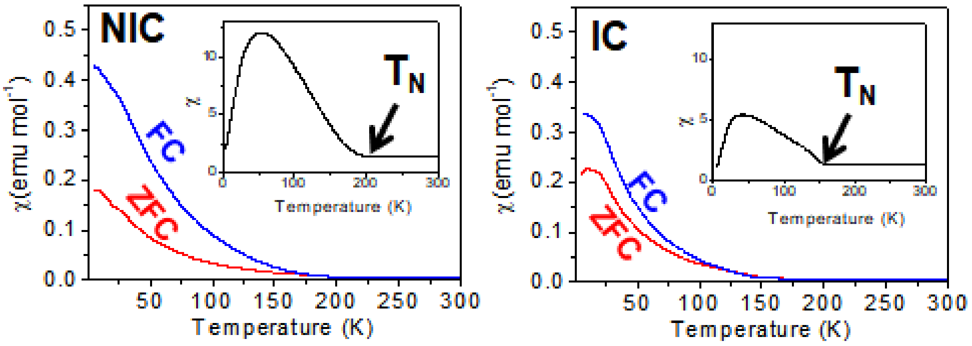

Figure S6. Magnetic properties of the IC and NIC sample. Temperature dependence of the molar magnetic susceptibility of the NIC and IC sample showing bifurcation of the FC and ZFC curves, indicating magnetic ordering in the compound

To directly measure the distribution of Ni/Mn and the degree of local disordering in the two samples, the magnetic properties of the two samples were measured because the magnetic properties strongly depend on the distribution of 3d transition metals such as Ni and Mn. In Co-free Li-rich materials, the monoclinic phase (Li_2MnO_3) shows unique magnetic property because the octahedral Mn^{4+}(d3) ions shares edges with Mn^{4+}(d3) octahedral, and a 90° degree Mn^{4+}(d3)-O-Mn^{4+}(d3) magnetic exchange interaction ($LiMn_6$-like region) is possible, which has strongly antiferromagnetic (AFM) property based on Goodenough's rule.[7] The two samples show quite different magnetic properties such as the Neel temperature (T_N), which shows the ordering of Mn in the materials, and the strength of bifurcation of FC(Field Cooled) and ZFC (Zero Field Cooled) curves, which is a direct measurement of the extent of AFM ordering.[8] In Figure S6, the IC sample shows much lower ordering transition temperature (T_N) and lower strength of FC and ZFC curve bifurcation (i.e., a narrower bifurcation) than the NIC sample. These results indicate that the magnetic ordering from $LiMn_6$-like region ($LiMn_6$ ordering) is decreased in the IC sample, even if both samples have the same TM oxidation state (Ni^{2+}, Mn^{4+} in Figure S8). Given that Li ions are distributed between the two phases and the charge balance should be met, the changed local structure by Li-Ni inter-diffusion with the increased cation disordering in the IC sample will lead to the decrease in the strength of the bifurcation of FC and ZFC curve and the decrease in the AFM ordering transition temperature.

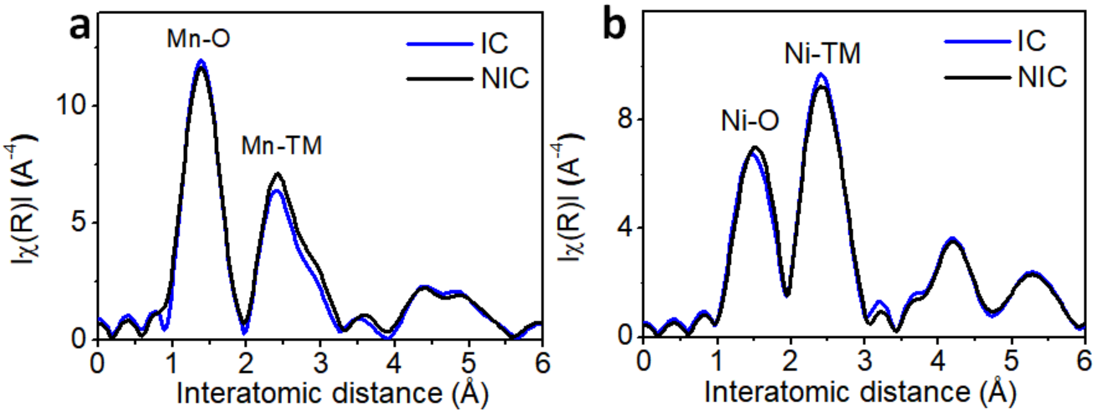

Figure S7. EXAFS data of of the two samples. Magnitude of the Fourier transformed Mn and Ni K-edge spectra. **a,** Mn K-edge EXAFS data and **b,** Ni K-edge EXAFS data of the NIC and IC sample

The metal–oxygen and metal–metal distances can be estimated from EXAFS spectra to understand the changes in the oxidation state of each transition metal and in their local environment.[9] Figure S7 shows metal–first oxygen neighbor distances and metal–first metal neighbor distances obtained from the refinement of k^3-weighted Fourier transforms of EXAFS spectra with the absolute error of ±0.01 (Table S5). Since changes in the intensity and position are related to the M-O and M-M distance, similar intensity and position observed in both NIC and IC sample (M = Mn, Ni) indicate that both samples have similar TM oxidation state but hard to distinguish the structural disorder.

However, the direct relevant structural parameter in EXAFS data is the Debye Waller factor, which can represent structural disorder of the central absorbing elements.[4b, 10] As seen Table S5, the IC sample has much higher mean-square disorder (σ^2) from the EXAFS Debye-Waller factor of M-O bond and M-M bond than the NIC sample. This, in combination with the increase of the mean-square disorder (σ^2) from of the M-O and M-M pair, reflects an increase of cation disordering in the structure after Li/TM inter-diffusion between phases in IC sample, which is consistent with Neutron Rietveld refinement result, HAADF-STEM and Li-NMR results of IC sample.

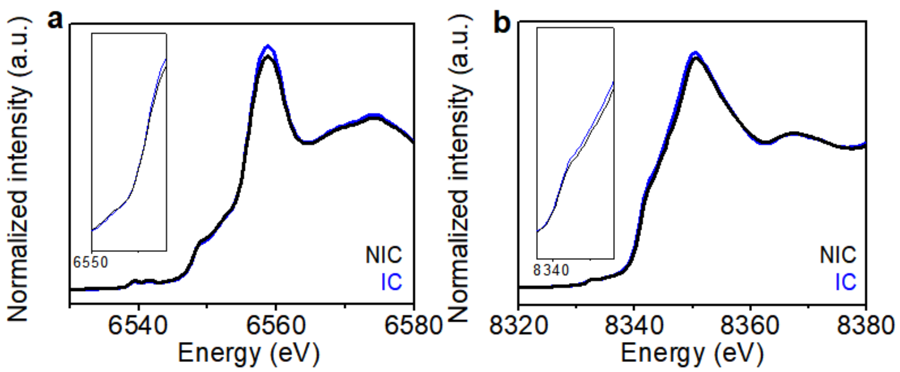

Figure S8. Mn and Ni K-edge of pristine samples. **a,** Mn K-edge XANES data and **b,** Ni K-edge XANES data of the NIC and IC sample, indicating similar Ni and similar Mn oxidation states in the samples. The Mn^{4+} peak (6560 eV) peaks in pristine NIC and IC sample is the same as that of Mn^{4+} (~ 6560 eV) [11], and the Ni^{2+} peak (8350 eV) in pristine NIC and IC sample is the same as that of Ni^{2+} (~ 8350 eV) [11]

- **DFT calculations of formation energies of the IC samples that have the Li-Ni inter-diffusion between the two layered phases**

DFT total energy was computed using the Vienna *ab initio* Simulation Package (VASP)[12] and the PBE functional in the GGA[13] and PAW method.[14] The on-site interaction is corrected by the Hubbard U parameters of 6.0 eV for the Ni and 3.9 eV for the Mn 3*d* orbitals.[15] Spin-polarized calculations were performed with a plane-wave energy cutoff = 520 eV and the *k*-points grid density > 0.01 *k*-points/Å^3. The lattice parameters and atomic positions were optimized until the energies converged to < 10^{-5} eV/atom and forces converged to < 10^{-3} eV/Å. The Li-Ni inter-diffusion in the IC sample increases the inter-diffused of cation (Li/TMs) from the composite of $LiNi_{0.5}Mn_{0.5}O_2$ (LNMO) and Li_2MnO_3 to the composite of $(Li_{1+2x}Ni_{0.5-x}Mn_{0.5})O_2$ (LNMO-like) and $(Li_{2-2y}Ni_yMn)O_3$ (Li_2MnO_3-like), by extracting *x* mole of Ni from LNMO and inserting the Ni into Li_2MnO_3, and simultaneously extracting 2*y* mole of Li from Li_2MnO_3 and inserting it into LNMO. To compute the total energy of these systems after the Li-Ni inter-diffusion, we modeled the $Li_{1+2x}Ni_{0.5-x}Mn_{0.5}O_2$ and $Li_{2-2y}Ni_yMnO_3$ phases separately using the layered and flower structures of LNMO,[16] and Li_2MnO_3 in the C2/m space group. We used supercells that contained 8 formula units (f.u.) of the LNMO layered phase, 12 f.u. of flower LNMO phase, and 16 f.u. of Li_2MnO_3. Amounts of the Li-Ni inter-diffusion $x = 0$, 0.125, and 0.25 were considered for layered $Li_{1+2x}Ni_{0.5-x}Mn_{0.5}O_2$; $x = 0$, 0.083, 0.167, and 0.25 for flower $Li_{1+2x}Ni_{0.5-x}Mn_{0.5}O_2$; and $y = 0$, 0.0625, 0.125, and 0.25 for $Li_{2-2y}Ni_yMnO_3$ considering the computational cell dimensions.

The Li/TMs distribution in the $Li_{1+2x}Ni_{0.5-x}Mn_{0.5}O_2$ and $Li_{2-2y}Ni_yMnO_3$ phases after Li-Ni inter-diffusion was initially predicted based on the electrostatic interaction energies, i.e. Ewald energies. For further treatment in DFT, we selected > 30 atomic configurations that had the lowest electrostatic energies for each structure and composition. We confirmed that in each structure and composition, all possible combinations of cation locations were included in our DFT calculations; for example, Ni in Li layers, Ni in TM layers, Li in Li layers, and Li in TM layers, if available. The atomic positions and cell parameters were further optimized within DFT, and the calculated DFT total energies of Li-Ni inter-diffused systems were plotted (Figure 2d) with respect to the energies before the Li/Ni inter-diffusion. The net formation energy of the Li-Ni inter-diffused system (Figure 2d, black filled squares) is marginally positive, < 0.22 eV per $Li_{1.2}Ni_{0.2}Mn_{0.6}O_2$ up to $x = 0.25$. Our calculations do not include configurational and thermal entropies that can further reduce the net formation energies, and may increase the accessibility of the composite structure during synthesis.

II. Anion redox reaction in both R phase and M phase of the IC sample with full Li extraction.

- The Ni K-edge and Mn K-edge of the IC electrodes with different states during 1 cycle using *ex-situ* XANES measurements

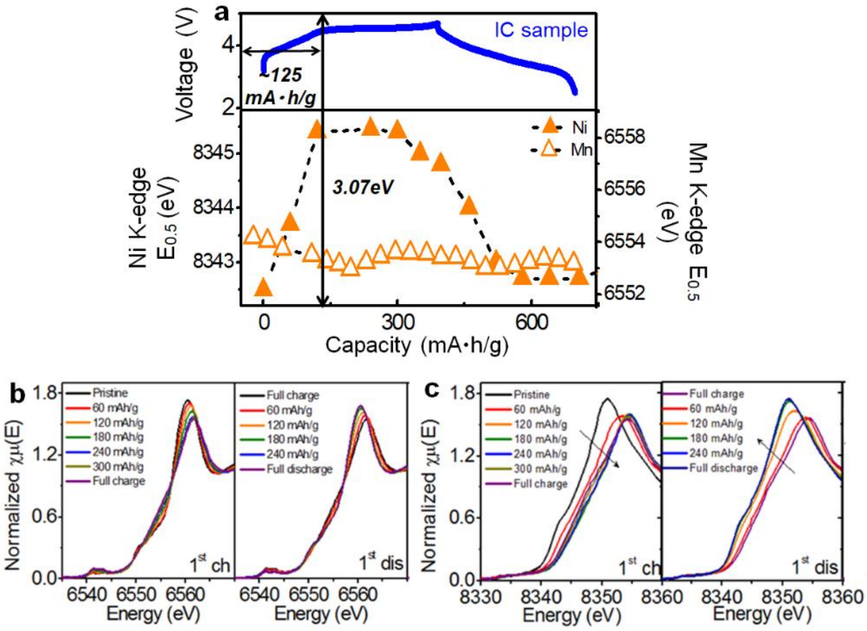

Figure S9. *Ex-situ* XANES measurements for the electrodes prepared at different electrochemical 1 cycle. a, Variations of half-edge position for Ni and Mn K-edge XANES spectra of the IC sample during 1st cycle. **b,** Normalized *ex-situ* XANES spectra of Mn K-edge during 1st cycle. **c,** Normalized *ex-situ* XANES spectra of Ni K-edge during 1st cycle. The TM redox reaction shows dominantly in Ni redox, whereas Mn redox is negligible; this result is consistent with the literature. (Ni^{2+} in pristine IC and NIC sample (8350 eV) is similar with reported Ni^{2+} (~8350 eV). Mn^{4+}(6560 eV) in pristine IC and NIC sample is similar with reported Mn^{4+} (~6560 eV). Ni^{4+} (8355 eV) in fully-charged IC and NIC sample is similar with reported Ni^{4+} (~8355 eV))[11, 17] Data pre-processing operations such as deglitching, energy calibration, normalization, and least square fitting with a theory were performed as described by Kelly et al. by using IFEFFIT which used the FEFF code.[18] All *ex-situ* XAS samples are prepared by galvanostatic charge/discharge at the state of charge/discharge per 60 mA·h/g.

- **The Ni K-edge, Mn K-edge and O K-edge of the electrodes during 1cycle using *ex-situ* SXAS measurements**

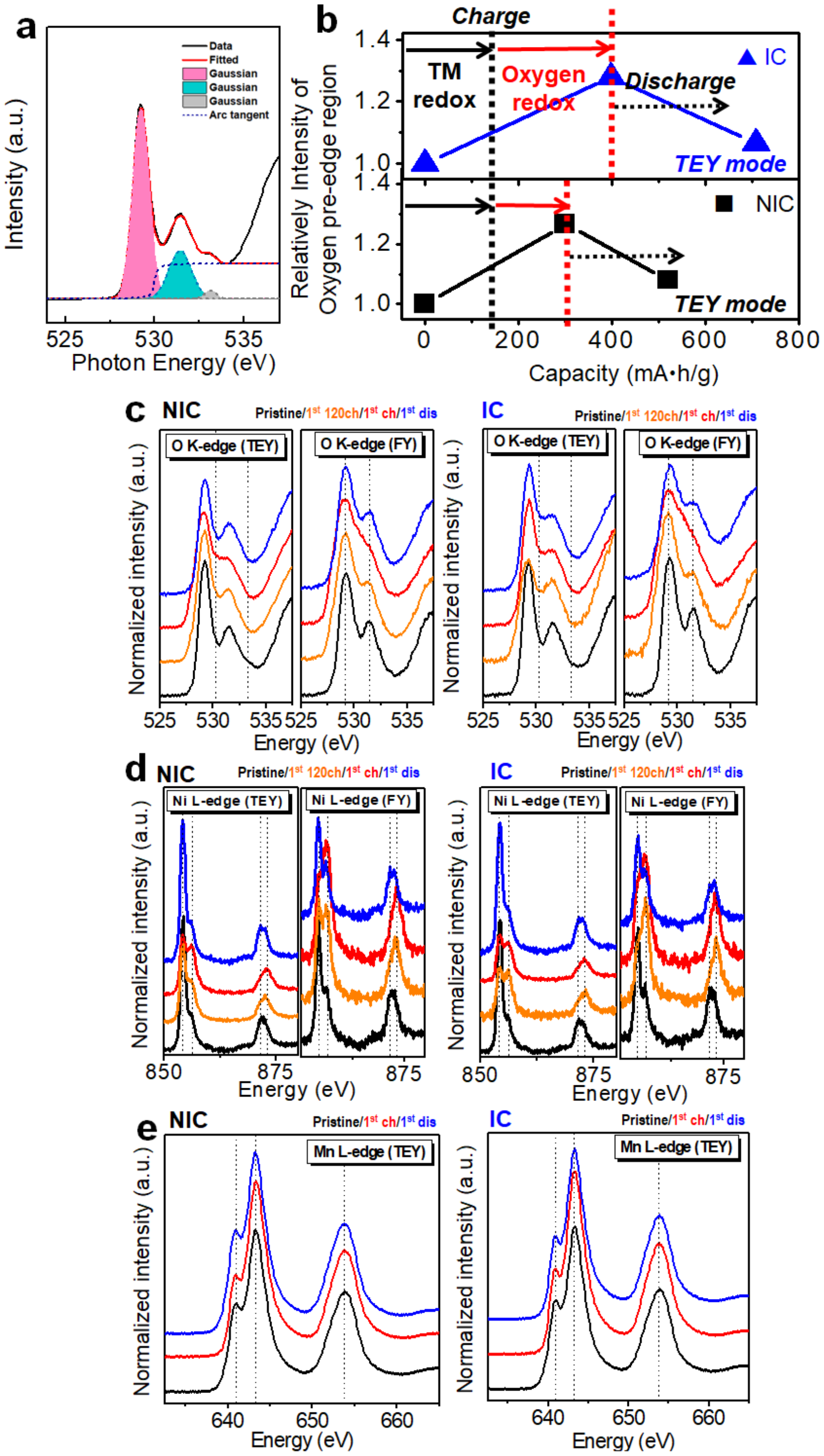

Figure S10. ***Ex-situ*** **Soft-XAS measurements for the electrodes prepared at different electrochemical 1 cycle. a,** Data pre-processing operations of Oxygen K-edge such as deglitching, energy calibration, normalization, and least square fitting with theory were

performed by using WINXAS program with two or three Gaussian functions and one Arc tangent function. **b,** Variation of integrated intensity of O k-edge in the low-energy region (< 534 eV) during 1st cycle in total electron yield (TEY, surface sensitive; filled) mode of the IC sample and the NIC sample (Table S6). Soft XAS spectra of pristine, full 1st charge and full 1st discharge for **c,** O K-edge in TEY and FY mode (pre-edge region < 534 eV) **d,** Ni L-edge in TEY and FY mode, **e,** Mn L-edge in TEY mode for NIC and IC sample.

- **The amount of Li with the electrodes with different states of charge (SOCs) using *ex-situ* NMR measurements**

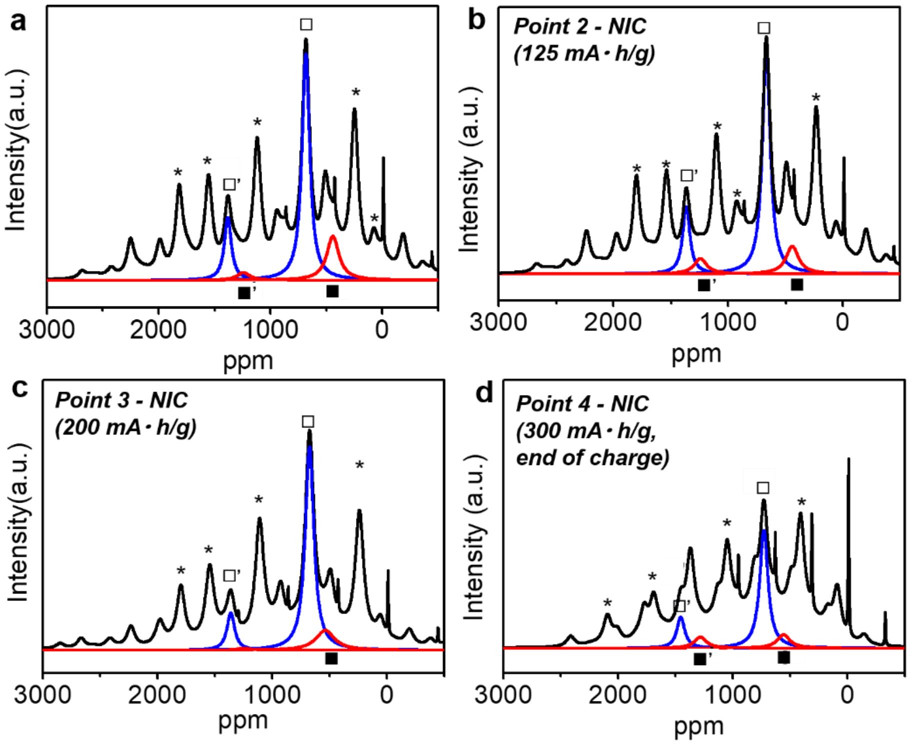

Figure S11. **^{7}Li MAS NMR spectra of the ex-situ electrodes of <u>the NIC sample</u> at MAS spinning speeds of 34 kHz of different SOCs, except for the end of charge (25 kHz) a,** pristine **b,** 110 mA·h/g **c,** 185 mA·h/g **d,** 300 mA·h/g (end of charge) (□: Li in Li layer of Li_2MnO_3, □′: Li in TM layer of Li_2MnO_3, ■: Li in Li layer of LNMO, ■′: Li in TM layer of LNMO, *: side bands) The NMR results are presented here as mol of Li, as deduced from normalized integrated intensities obtained during charge/discharge, considering that the integrated intensity of the lithiated sample (pristine sample) contains 100% Li (i.e., 1.2 mol Li).

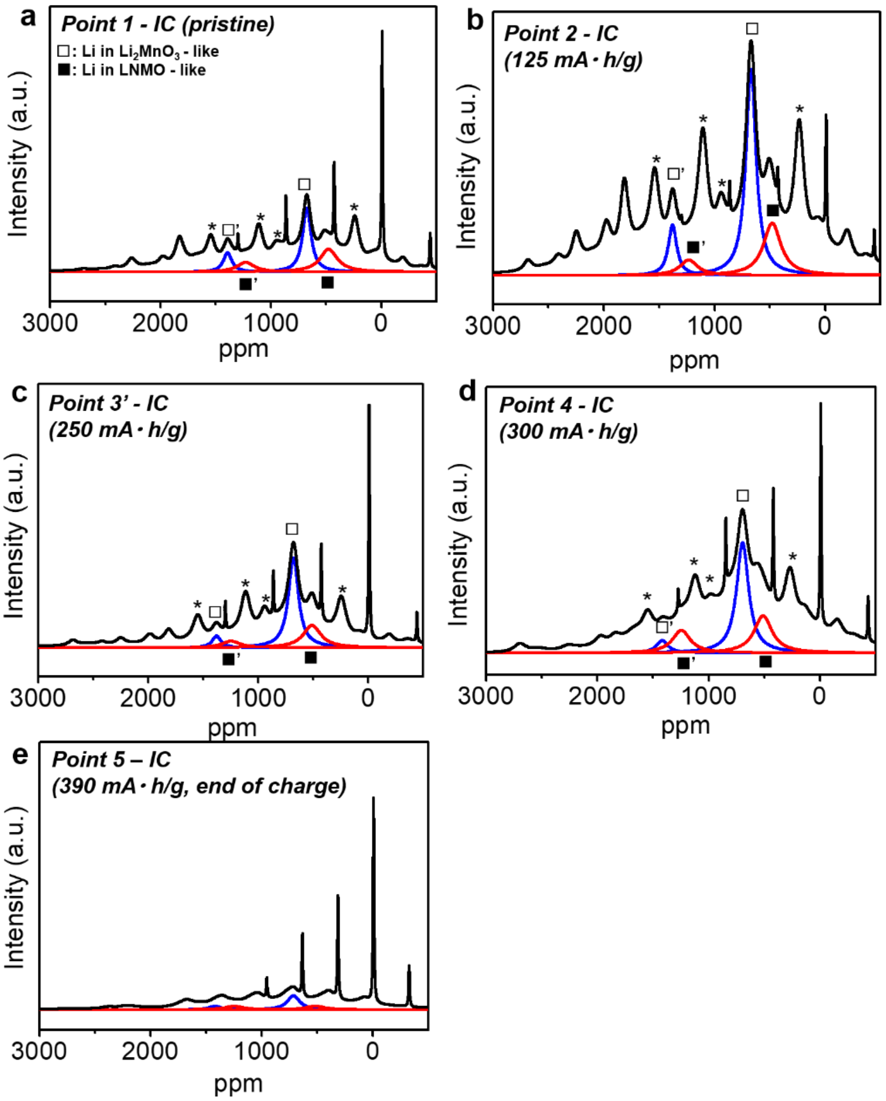

Figure S12. **^{7}Li MAS NMR spectra of the ex-situ electrodes of <u>the IC sample</u> at MAS spinning speeds of 34 kHz of different State-of-charges (SOCs), except for the end of charge (25 kHz) a,** pristine **b,** 125 mA·h/g **c,** 250 mA·h/g **d,** 300 mA·h/g **e,** 380 mA·h/g (end of charge) (□: Li in Li layer of Li_2MnO_3-like, □′: Li in TM layer of Li_2MnO_3-like, ■: Li in Li layer of LNMO-like, ■′: Li in TM layer of LNMO-like, *: side bands) The NMR results are presented as mol of Li, as deduced from normalized integrated intensities obtained during charge/discharge, considering that the integrated intensity of the lithiated (pristine) sample contains 100% Li (i.e., 1.2 mol Li).

- **DFT-predicted density of states in Li-excess LNMO using the Heyd–Scuseria–Ernzerhof (HSE) hybrid functional**

In the Li-Ni inter-diffused system, the $Li_{2-2x}Ni_xMnO_3$ region gains an additional redox center upon insertion of Ni^{2+}, because in theory it can be oxidized up to Ni^{4+}, with consequent increase in the contribution of this phase to the net capacity of the system. In contrast, the $Li_{1+2x}Ni_{0.5-x}Mn_{0.5}O_2$ phase loses Ni^{2+} ions. Therefore, to increase net capacity by using the Li-Ni inter-diffusion strategy, the availability and reversibility of oxygen oxidation from O^{2-} to O^{x-} ($x < 2$) or O_2^{y-} ($y \leq 2$) must be verified.
The relative positions of TM 3d and O 2p orbital bands may determine the contribution of oxygen oxidation to the capacity, so we performed DFT calculations to investigate the electronic density of states (DOS) of $Li_{1+2x}Ni_{0.5-x}Mn_{0.5}O_2$. To represent the energy band positions more reliably than the GGA calculations can, we used the HSE screened hybrid functional[19] and applied it to the most stable structures predicted in Figure 2c. The atomic positions and lattice parameters were re-optimized within HSE, and the pseudopotential, plane-wave energy cutoff, *k*-points grid, and energy and force convergence criteria were the same as in the GGA calculations.
The DOS of $Li_{1+2x}Ni_{0.5-x}Mn_{0.5}O_2$ near to the Fermi level were predicted for $x = 0$, 0.125, and 0.25 (Figure S13). In all three systems, the shapes of Ni 3d (Figure S13, red solid lines) and O 2p orbital-projected DOS (Figure S13, black solid lines) resemble each other; therefore, we conclude that the valence band maximum (VBM) is dominated by Ni-O hybridization. Meanwhile, hybridization of Mn 3d and O 2p is responsible for conduction band minimum (CBM) (not shown). To assess how each energy band contributes to electron extraction from $Li_{1+2x}Ni_{0.5-x}Mn_{0.5}O_2$ upon charging, we adopted rigid-band theory and calculated Fermi level shifts for electron-deficient systems. To model charge-balanced systems when all Li^+ ions are removed from $Li_{1+2x}Ni_{0.5-x}Mn_{0.5}O_2$, a total of 1, 1.25, or 1.5 electrons per f.u. were extracted for each of $x = 0$, 0.125, and 0.25. The Fermi levels shifted (Figure S13). In $LiNi_{0.5}Mn_{0.5}O_2$ and $Li_{1.25}Ni_{0.375}Mn_{0.5}O_2$, the Ni 3d and O 2p bands look alike from the shifted Fermi levels, so we infer that the charging process is charge-balanced by electrons donated from the Ni 3d-O 2p hybridized band (Figure S13a, b, dashed vertical lines) to the Fermi levels before delithiation (Figure S13a, b, *x*-axis value = 0).
Electron donation from O ions can be observed from DOS in $Li_{1.5}Ni_{0.25}Mn_{0.5}O_2$. The shifted Fermi level in $Li_{1.5}Ni_{0.25}Mn_{0.5}O_2$ (Figure S13c, dashed vertical lines) is located in the regime where the shape of the O 2p DOS is distinct from the shape of the Ni 3d DOS. The distinct

shapes of O 2p DOS from Ni 3d and Mn 3d DOS near the shifted Fermi level can be understood as appearance of the labile O 2p orbitals.[20] Our electronic structure calculations confirm that the Li-Ni inter-diffusion promotes oxygen oxidation in the $Li_{1+2x}Ni_{0.5-x}Mn_{0.5}O_2$ regime, and partly explains the increased capacity in the overall system.

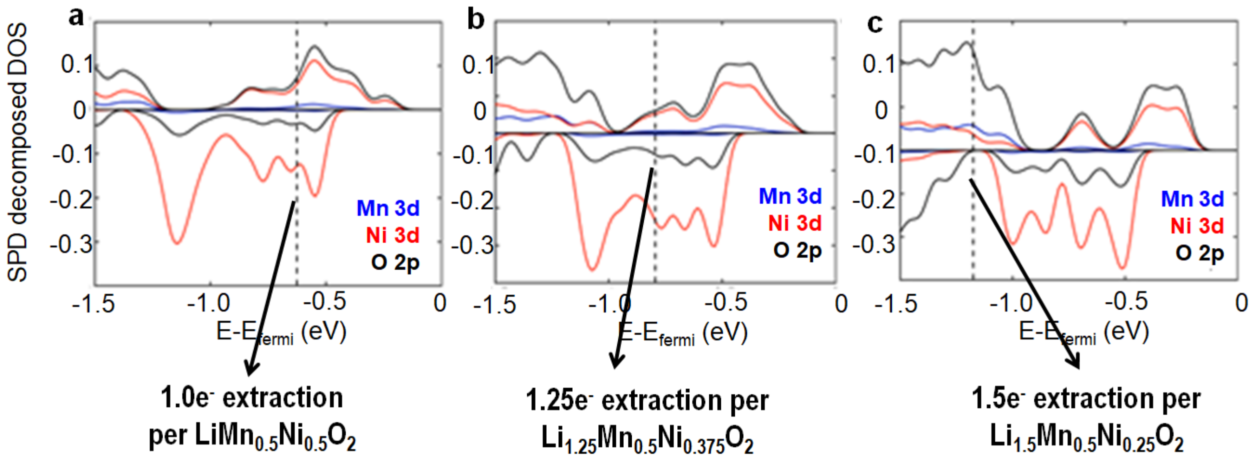

Figure S13. Atom and orbital projected density of states (pDOS) a, $LiNi_{0.5}Mn_{0.5}O_2$ **b,** $Li_{1.25}Ni_{0.375}Mn_{0.5}O_2$ and **c,** $Li_{1.5}Ni_{0.25}Mn_{0.5}O_2$, representing the Li-Ni inter-diffused $LiNi_{0.5}Mn_{0.5}O_2$ phase after 0, 0.125, and 0.25 mole of Ni extraction. The energy is set with respect to the Fermi level before electron extraction. Blue: Mn 3d, red: Ni 3d, black: O 2p orbital projected DOS. Black dashed vertical lines: Fermi level based on the rigid band assumption when all Li^+ ions and corresponding number of electrons are extracted.

III. Electrochemical performance of the samples

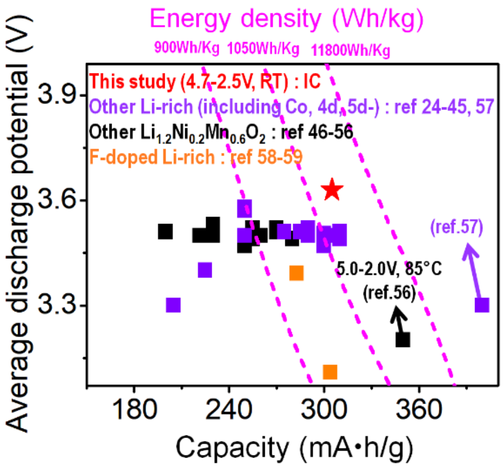

Figure S14. Average discharge potential vs. discharge capacity with energy density plot among Co-free $Li_{1.2}Ni_{0.2}Mn_{0.6}O_2$ compounds (including IC sample and NIC sample), and other Li-rich materials exhibiting the highest first discharge capacity and average potential.[21] All electrochemical tests of IC sample and NIC sample were performed at room temperature and the operating voltage window was from 2.5 V to 4.7 V.

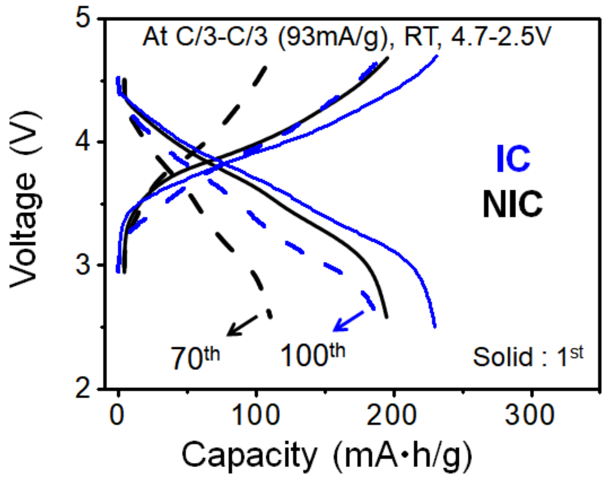

Figure S15. Voltage profiles at C/3 charge and discharge rate during cycles for the NIC and IC samples. Electrochemical tests were performed at room temperature and the operating voltage window was from 2.5 V to 4.7 V. Average potential is defined as the potential obtained at half discharge capacity or at half energy density during discharge.

Table S1: Nominal composition and Real composition (from ICP) for each samples

Nominal composition	$Li_{1.2}Ni_{0.2}Mn_{0.6}O_2$	
Real composition (from ICP)	IC	NIC
Li	1.22	1.22
Ni	0.194	0.195
Mn	0.6	0.6

Table S2: Rietveld refinement results of neutron powder diffraction (NPD) measurements for the IC sample and the NIC sample. (Site Occupancies of the metal atoms in the Li, TM Layer of $(Li_{1+2x}Ni_{1-x}Mn_{0.5})O_2$ and $(Li_{2-2y}Ni_yMn)O_3$ of IC and NIC sample and agreement factors, as determined by the Rietveld refinement of NPD using the two-phase model, i.e., a combination of $LiTMO_2$ (R-3m) phase, and Li_2MnO_3 (C/2m) phase)

Refined parameters		Sample name	
		NIC	IC
LNMO (R-3m)	a	2.8689(5)	2.8600(5)
	c	14.2702(2)	14.2566(3)
Li_2MnO_3 (C2/m)	a	4.9474(4)	4.9534(5)
	b	8.5522(2)	8.5637(8)
	c	5.0240(5)	5.0346(3)
	beta	109.101(1)	109.337(8)
Atom occupancies (Li/Ni) : $(Li_{1+2x}Ni_{0.5-x}Mn_{0.5})O_2$	Li layer (3a)	0.943(2)/0.057(2)	0.950(1)/0.050(1)
	TM layer (3b)	0.057(2)/0.443(2)	0.214(1)/0.285(1)
	Li in interstitial site (36i)	0.0	0.166
Atom position (x, y, z) : $(Li_{1+2x}Ni_{0.5-x}Mn_{0.5})O_2$	Li layer (3a)	(0, 0, 0)	(0, 0, 0)
	TM layer (3b)	(0, 0, 0.5)	(0, 0, 0.5)
	Li in interstitial site (36i)		(0.083, 0.416, 0.228)
B factor (Å^2)	Li layer (3a)	0.74	0.91
	TM layer (3b)	1.31	0.82
Atom occupancies (Li/Ni) : $(Li_{2-2y}Ni_yMn)O_3$	TM layer (2b)	0.5/0.0	0.374(6)/0.048(6)
	Li layer (4h)	1.0/0.0	0.844(8)/0.094(8)
Atom position (x, y, z) : $(Li_{2-2y}Ni_yMn)O_3$	TM layer (2b)	(0, 0.5, 0)	(0, 0.5, 0)
	Li layer (4h)	(0, 0.678, 0)	(0, 0.655, 0)
B factor (Å^2)	TM layer (2b)	1.15	1.55
	Li layer (4h)	1.51	1.74

wt% of R phase/M phase (LNMO/Li_2MnO_3)		36/64	40/60
Agreement factors	R_p R_{wp},%	2.79 3.60	2.77 3.53
	$\times^2$	1.96	2.10

Table S3: Rietveld refinement results of synchrotron X-ray diffraction (SXRD) measurements for the IC sample and the NIC sample. (Site Occupancies of the metal atoms in the Li, TM Layer of $(Li_{1+2x}Ni_{1-x}Mn_{0.5})O_2$ and $(Li_{2-2y}Ni_yMn)O_3$ of IC and NIC sample and agreement factors, as determined by the Rietveld refinement of SXRD using the two-phase model, i.e., a combination of $LiTMO_2$ (R-3m) phase, and Li_2MnO_3 (C/2m) phase)

Refined parameters		Sample name	
		NIC	IC
LNMO (R-3m)	a	2.8645(3)	2.8578(4)
	c	14.2792(1)	14.2564(2)
Li_2MnO_3 (C2/m)	a	5.0359(4)	5.0047(5)
	b	8.5250(2)	8.5589(3)
	c	5.0135(1)	5.0461(2)
	beta	110.001(1)	109.223(7)
Atom occupancies (Li/Ni) : $(Li_{1+2x}Ni_{0.5-x}Mn_{0.5})O_2$	Li layer (3a)	0.938(3)/0.062(3)	0.930(1)/0.070(1)
	TM layer (3b)	0.062(3)/0.438(3)	0.237(1)/0.263(1)
B factor (Å^2)	Li layer (3a)	0.88	0.83
	TM layer (3b)	0.71	0.76
Atom occupancies (Li/Ni) : $(Li_{2-2y}Ni_yMn)O_3$	TM layer (2b)	0.5/0.0	0.390(4)/0.031(4)
	Li layer (4h)	1.0/0.0	0.828(2)/0.102(2)
Atom position (x, y, z) : $(Li_{2-2y}Ni_yMn)O_3$	TM layer (2b)	(0, 0.5, 0)	(0, 0.5, 0)
	Li layer (4h)	(0, 0.698, 0)	(0, 0.672, 0)
B factor (Å^2)	TM layer (2b)	0.79	0.83
	Li layer (4h)	0.92	0.96
Agreement factors	R_p R_{wp},%	3.82 4.11	3.54 3.98
	$\times^2$	4.66	4.15

Table S4: Peak position δ [ppm], and relative intensity *I* [%] of the deconvoluted lines in ^{6}Li NMR spectra of the samples prepared in different experimental conditions

^{6}Li-NMR	IC		NIC	
	δ(ppm)	*I*(%)	δ(ppm)	*I*(%)
Li_{Li} in Li_2MnO_3(C2/m)	698	47	709	55
Li_{TM} in Li_2MnO_3(C2/m)	1419	11	1426	18
Li_{Li} in LNMO (R-3m)	535	22	529	18
Li_{TM} in LNMO (R-3m)	1288	9	1286	8
$Li_{interstitial}$ in LNMO (R-3m)	865	11	867	1

Table S5: Evolution of interatomic distance and Debye–Waller factors of M-O and M-M bond from EXAFS data of NIC and IC samples.

Sample	X-Y pair	R (Å)	σ^2 (10^{-3}Å^2)	R factor	X-Y pair	R (Å)	σ^2 (10^{-3}Å^2)	R factor
NIC	Mn-O	1.86(1)	1.3(10)	0.00835	Ni-O	2.01(1)	5.7(19)	0.00184
	Mn-M	2.82(1)	8.6(13)		Ni-M	2.87(1)	5.3(16)	
IC	Mn-O	1.88(1)	3.8(10)	0.00948	Ni-O	2.023(7)	5.4(13)	0.00127
	Mn-M	2.85(1)	9.6(12)		Ni-M	2.879(7)	6.0(12)	

Table S6: The values of the integrated pre-edge (< 534 eV) intensity of O K-edge soft XAS for the IC sample and the NIC sample for (a) FY mode and (b) TEY mode. The integrated intensities are obtained by fitting the spectra with Gaussian + arctangent fit functions. Total: calculated value, normalized: each calculated value divided by pristine.

**a** Total integrated pre-edge intensity (FY mode)	IC	NIC
	Normalized	Normalized
Pristine	1.000	1.000
1st 120mA·h/g charge	1.259	1.180
Full 1st charge	1.310	1.280
Full 1st discharge	1.100	1.143

**b** Total integrated pre-edge intensity (TEY mode)	IC	NIC
	Normalized	Normalized
Pristine	1.0000	1.0000
Full 1st charge	1.2781	1.2402
Full 1st discharge	0.9752	1.0796

IV. References

[1] a) A. Boulineau, L. Simonin, J.-F. o. Colin, E. Canévet, L. Daniel, S. b. Patoux, *Chemistry of Materials* **2012**, 24, 3558; b) D. Mohanty, A. Huq, E. A. Payzant, A. S. Sefat, J. Li, D. P. Abraham, D. L. Wood III, C. Daniel, *Chemistry of Materials* **2013**, 25, 4064.
[2] E. Kartini, M. Manawan, M. F. Collins, M. Avdeev, *Physica B: Condensed Matter* **2018**, 551, 320.
[3] a) J. Bréger, Y. S. Meng, Y. Hinuma, S. Kumar, K. Kang, Y. Shao-Horn, G. Ceder, C. P. Grey, *Chemistry of Materials* **2006**, 18, 4768; b) P. Manikandan, M. Ananth, T. P. Kumar, M. Raju, P. Periasamy, K. Manimaran, *journal of Power Sources* **2011**, 196, 10148.
[4] a) J. Zheng, M. Gu, A. Genc, J. Xiao, P. Xu, X. Chen, Z. Zhu, W. Zhao, L. Pullan, C. Wang, *Nano letters* **2014**, 14, 2628; b) X. Yu, Y. Lyu, L. Gu, H. Wu, S. M. Bak, Y. Zhou, K. Amine, S. N. Ehrlich, H. Li, K. W. Nam, *Advanced Energy Materials* **2014**, 4, 1300950; c) Y. Zuo, B. Li, N. Jiang, W. Chu, H. Zhang, R. Zou, D. Xia, *Advanced Materials* **2018**, 30, 1707255.
[5] a) J. Bréger, M. Jiang, N. Dupré, Y. S. Meng, Y. Shao-Horn, G. Ceder, C. P. Grey, *Journal of Solid State Chemistry* **2005**, 178, 2575; b) R. Stoyanova, S. Ivanova, E. Zhecheva, A. Samoson, S. Simova, P. Tzvetkova, A.-L. Barra, *Physical Chemistry Chemical Physics* **2014**, 16, 2499.
[6] a) C. P. Grey, N. Dupré, *Chemical Reviews* **2004**, 104, 4493; b) K. Chiba, M. Shikano, H. Sakaebe, *RSC Advances* **2018**, 8, 26335; c) F. Dogan, J. R. Croy, M. Balasubramanian, M. D. Slater, H. Iddir, C. S. Johnson, J. T. Vaughey, B. Key, *Journal of The Electrochemical Society* **2015**, 162, A235.
[7] J. B. Goodenough, *Physical Review* **1960**, 117, 1442.
[8] D. Mohanty, A. S. Sefat, J. Li, R. A. Meisner, A. J. Rondinone, E. A. Payzant, D. P. Abraham, D. L. Wood III, C. Daniel, *Physical Chemistry Chemical Physics* **2013**, 15, 19496.
[9] T. Kim, B. Song, A. J. Lunt, G. Cibin, A. J. Dent, L. Lu, A. M. Korsunsky, *Chemistry of Materials* **2016**, 28, 4191.
[10] Y. Tsai, B. Hwang, G. Ceder, H. Sheu, D. Liu, J. Lee, *Chemistry of materials* **2005**, 17, 3191.
[11] a) K. Luo, M. R. Roberts, R. Hao, N. Guerrini, D. M. Pickup, Y.-S. Liu, K. Edström, J. Guo, A. V. Chadwick, L. C. Duda, *Nature chemistry* **2016**, 8, 684; b) M. Oishi, C. Yogi, I. Watanabe, T. Ohta, Y. Orikasa, Y. Uchimoto, Z. Ogumi, *Journal of Power Sources* **2015**, 276, 89.
[12] G. Kresse, J. Furthmüller, *Physical review B* **1996**, 54, 11169.
[13] J. P. Perdew, K. Burke, M. Ernzerhof, *Physical review letters* **1996**, 77, 3865.
[14] P. E. Blöchl, *Physical review B* **1994**, 50, 17953.
[15] P. Giannozzi, S. Baroni, N. Bonini, M. Calandra, R. Car, C. Cavazzoni, D. Ceresoli, G. L. Chiarotti, M. Cococcioni, I. Dabo, *Journal of physics: Condensed matter* **2009**, 21, 395502.
[16] Y. Hinuma, Y. S. Meng, K. Kang, G. Ceder, *Chemistry of Materials* **2007**, 19, 1790.
[17] H. Koga, L. Croguennec, M. Ménétrier, P. Mannessiez, F. Weill, C. Delmas, S. Belin, *The Journal of Physical Chemistry C* **2014**, 118, 5700.
[18] A. Athena, *Journal of Synchrotron Radiation* **2005**, 12, 537.
[19] J. Heyd, G. E. Scuseria, M. Ernzerhof, *The Journal of chemical physics* **2003**, 118, 8207.
[20] D.-H. Seo, J. Lee, A. Urban, R. Malik, S. Kang, G. Ceder, *Nature chemistry* **2016**, 8, 692.

[21] a) N. Yabuuchi, M. Takeuchi, M. Nakayama, H. Shiiba, M. Ogawa, K. Nakayama, T. Ohta, D. Endo, T. Ozaki, T. Inamasu, *Proceedings of the National Academy of Sciences* **2015**, 112, 7650; b) P. E. Pearce, A. J. Perez, G. Rousse, M. Saubanère, D. Batuk, D. Foix, E. McCalla, A. M. Abakumov, G. Van Tendeloo, M.-L. Doublet, *Nature Materials* **2017**, 16, 580; c) M. Sathiya, K. Ramesha, G. Rousse, D. Foix, D. Gonbeau, A. Prakash, M. Doublet, K. Hemalatha, J.-M. Tarascon, *Chemistry of Materials* **2013**, 25, 1121; d) M. Sathiya, G. Rousse, K. Ramesha, C. Laisa, H. Vezin, M. T. Sougrati, M.-L. Doublet, D. Foix, D. Gonbeau, W. Walker, *Nature materials* **2013**, 12, 827; e) X. Zhang, I. Belharouak, L. Li, Y. Lei, J. W. Elam, A. Nie, X. Chen, R. S. Yassar, R. L. Axelbaum, *Advanced Energy Materials* **2013**, 3, 1299; f) H. Liu, C. Du, G. Yin, B. Song, P. Zuo, X. Cheng, Y. Ma, Y. Gao, *Journal of Materials Chemistry A* **2014**, 2, 15640; g) P. Remith, N. Kalaiselvi, *Nanoscale* **2014**, 6, 14724; h) S. Guo, H. Yu, P. Liu, X. Liu, M. Chen, M. Ishida, H. Zhou, *Journal of Materials Chemistry A* **2014**, 2, 4422; i) J. Liu, M. Hou, J. Yi, S. Guo, C. Wang, Y. Xia, *Energy & Environmental Science* **2014**, 7, 705; j) Q. Li, G. Li, C. Fu, D. Luo, J. Fan, L. Li, *ACS applied materials & interfaces* **2014**, 6, 10330; k) L. Guo, N. Zhao, J. Li, C. He, C. Shi, E. Liu, *ACS applied materials & interfaces* **2014**, 7, 391; l) Y. Chen, K. Xie, C. Zheng, Z. Ma, Z. Chen, *ACS applied materials & interfaces* **2014**, 6, 16888; m) B. Qiu, J. Wang, Y. Xia, Z. Wei, S. Han, Z. Liu, *ACS applied materials & interfaces* **2014**, 6, 9185; n) J. Li, J. Camardese, R. Shunmugasundaram, S. Glazier, Z. Lu, J. Dahn, *Chemistry of Materials* **2015**, 27, 3366; o) F. Fu, Y.-P. Deng, C.-H. Shen, G.-L. Xu, X.-X. Peng, Q. Wang, Y.-F. Xu, J.-C. Fang, L. Huang, S.-G. Sun, *Electrochemistry Communications* **2014**, 44, 54; p) S. Han, B. Qiu, Z. Wei, Y. Xia, Z. Liu, *Journal of Power Sources* **2014**, 268, 683; q) O. Toprakci, H. A. Toprakci, Y. Li, L. Ji, L. Xue, H. Lee, S. Zhang, X. Zhang, *Journal of Power Sources* **2013**, 241, 522; r) M. Bettge, Y. Li, B. Sankaran, N. D. Rago, T. Spila, R. T. Haasch, I. Petrov, D. P. Abraham, *Journal of Power Sources* **2013**, 233, 346; s) S. Shi, J. Tu, Y. Tang, Y. Yu, Y. Zhang, X. Wang, C. Gu, *Journal of Power Sources* **2013**, 228, 14; t) J. Zheng, S. Deng, Z. Shi, H. Xu, H. Xu, Y. Deng, Z. Zhang, G. Chen, *Journal of power sources* **2013**, 221, 108; u) W. Choi, A. Benayard, J.-H. Park, J. Park, S.-G. Doo, J. Mun, *Electrochimica Acta* **2014**, 117, 492; v) S. Shi, J. Tu, Y. Zhang, Y. Zhang, X. Zhao, X. Wang, C. Gu, *Electrochimica Acta* **2013**, 108, 441; w) Y. Li, Y. Bai, C. Wu, J. Qian, G. Chen, L. Liu, H. Wang, X. Zhou, F. Wu, *Journal of Materials Chemistry A* **2016**, 4, 5942; x) Y. Li, Y. Bai, X. Bi, J. Qian, L. Ma, J. Tian, C. Wu, F. Wu, J. Lu, K. Amine, *ChemSusChem* **2016**; y) L. Li, L. Wang, X. Zhang, Q. Xue, L. Wei, F. Wu, R. Chen, *ACS Applied Materials & Interfaces* **2017**; z) J. Zhang, H. Zhang, R. Gao, Z. Li, Z. Hu, X. Liu, *Physical Chemistry Chemical Physics* **2016**, 18, 13322; aa) H. Liu, D. Qian, M. G. Verde, M. Zhang, L. Baggetto, K. An, Y. Chen, K. J. Carroll, D. Lau, M. Chi, *ACS applied materials & interfaces* **2015**, 7, 19189; ab) X. He, J. Wang, L. Wang, J. Li, *Materials* **2016**, 9, 661; ac) Y. Liu, Q. Wang, Z. Zhang, A. Dou, J. Pan, M. Su, *Advanced Powder Technology* **2016**, 27, 1481; ad) Y. Zang, C.-X. Ding, X.-C. Wang, Z.-Y. Wen, C.-H. Chen, *Electrochimica Acta* **2015**, 168, 234; ae) Y. Zang, X. Sun, Z.-F. Tang, H.-F. Xiang, C.-H. Chen, *RSC Advances* **2016**, 6, 30194; af) K. Luo, M. R. Roberts, N. Guerrini, N. Tapia-Ruiz, R. Hao, F. Massel, D. M. Pickup, S. Ramos, Y.-S. Liu, J. Guo, *Journal of the American Chemical Society* **2016**, 138, 11211; ag) T. Ohzuku, M. Nagayama, K. Tsuji, K. Ariyoshi, *Journal of Materials chemistry* **2011**, 21, 10179; ah) Y. Zuo, B. Li, N. Jiang, W. Chu, H. Zhang, R. Zou, D. Xia, *Advanced Materials* **2018**; ai) J. Lee, D. A. Kitchaev, D.-H. Kwon, C.-W. Lee, J. K. Papp, Y.-S. Liu, Z. Lun, R. J. Clément, T. Shi, B. D. McCloskey, *Nature* **2018**, 556, 185; aj) R. A. House, L. Jin, U. Maitra, K. Tsuruta, J. W. Somerville, D. P.

Förstermann, F. Massel, L. Duda, M. R. Roberts, P. G. Bruce, *Energy & Environmental Science* **2018**, 11, 926.
